# The Applications of Large Language Models in Mental Health: Scoping Review

**DOI:** 10.2196/69284

**Published:** 2025-05-05

**Authors:** Yu Jin, Jiayi Liu, Pan Li, Baosen Wang, Yangxinyu Yan, Huilin Zhang, Chenhao Ni, Jing Wang, Yi Li, Yajun Bu, Yuanyuan Wang

**Affiliations:** 1 Department of Statistics Faculty of Arts and Sciences Beijing Normal University Beijing China; 2 School of Psychology, Center for Studies of Psychological Application, and Guangdong Key Laboratory of Mental Health and Cognitive Science Key Laboratory of Brain, Cognition and Education Sciences, Ministry of Education South China Normal University Guangzhou, Guangdong China; 3 School of Statistics Beijing Normal University Beijing China; 4 Faculty of Computer Science Guangdong Polytechnic Normal University Guangzhou, Guangdong China; 5 The People’s Hospital of Pingbian County Honghe, Yunnan China

**Keywords:** mental health, large language models, application, process, performance, comparison

## Abstract

**Background:**

Mental health is emerging as an increasingly prevalent public issue globally. There is an urgent need in mental health for efficient detection methods, effective treatments, affordable privacy-focused health care solutions, and increased access to specialized psychiatrists. The emergence and rapid development of large language models (LLMs) have shown the potential to address these mental health demands. However, a comprehensive review summarizing the application areas, processes, and performance comparisons of LLMs in mental health has been lacking until now.

**Objective:**

This review aimed to summarize the applications of LLMs in mental health, including trends, application areas, performance comparisons, challenges, and prospective future directions.

**Methods:**

A scoping review was conducted to map the landscape of LLMs’ applications in mental health, including trends, application areas, comparative performance, and future trajectories. We searched 7 electronic databases, including Web of Science, PubMed, Cochrane Library, IEEE Xplore, Weipu, CNKI, and Wanfang, from January 1, 2019, to August 31, 2024. Studies eligible for inclusion were peer-reviewed articles focused on LLMs’ applications in mental health. Studies were excluded if they (1) were not peer-reviewed or did not focus on mental health or mental disorders or (2) did not use LLMs; studies that used only natural language processing or long short-term memory models were also excluded. Relevant information on application details and performance metrics was extracted during the data charting of eligible articles.

**Results:**

A total of 95 articles were drawn from 4859 studies using LLMs for mental health tasks. The applications were categorized into 3 key areas: screening or detection of mental disorders (67/95, 71%), supporting clinical treatments and interventions (31/95, 33%), and assisting in mental health counseling and education (11/95, 12%). Most studies used LLMs for depression detection and classification (33/95, 35%), clinical treatment support and intervention (14/95, 15%), and suicide risk prediction (12/95, 13%). Compared with nontransformer models and humans, LLMs demonstrate higher capabilities in information acquisition and analysis and efficiently generating natural language responses. Various series of LLMs also have different advantages and disadvantages in addressing mental health tasks.

**Conclusions:**

This scoping review synthesizes the applications, processes, performance, and challenges of LLMs in the mental health field. These findings highlight the substantial potential of LLMs to augment mental health research, diagnostics, and intervention strategies, underscoring the imperative for ongoing development and ethical deliberation in clinical settings.

## Introduction

### Background

Mental health is a growing global public issue, impacting nearly a billion people worldwide, with an estimated 1 in 7 adolescents affected [1,2]. Nevertheless, >70% of individuals with mental health disorders lack access to essential support and services [3]. Furthermore, >720,000 people commit suicide annually, with nearly three-quarters of these suicides occurring in low- and middle-income countries [4]. Consequently, there is an urgent need in mental health to facilitate efficient detection from large-scale data; deliver effective treatments and interventions to large populations; and ensure private, affordable health care and increased access to specialized psychiatrists. To address inadequate access to effective and equitable mental health care, large-scale, innovative solutions are imperative.

Large language models (LLMs), emerging in 2019, are advanced natural language processing (NLP) models capable of analyzing vast textual data and generating humanlike language [5]. Notable LLMs such as GPT-3/4 [6], Pathways Language Model (PaLM) [7], and LLaMA [8], constitute a category of foundational models, each with billions of parameters, trained on extensive textual data [9]. Using the transformer architecture and self-supervised pretraining, LLMs are adept at tackling a variety of NLP tasks, including information extraction, interaction, content generation, and logical reasoning [10]. In comparison to prior NLP models [11,12], LLMs exhibit superior performance in computational efficiency, large-scale data analyses, interaction, and external validity and applicability [9]. Furthermore, LLMs can be fine-tuned to cater to specific domains, including mental health, thereby empowering them to engage in natural language interactions and accomplish mental health–related tasks. LLMs would help address insufficient mental health care system capacity and provide efficient or personalized treatments. Therefore, the application of LLMs in mental health is expanding across diverse domains [13-16].

### Objective

Researchers have explored the applications of LLMs in mental health in various areas, encompassing screening or detecting mental disorders [17-19], supporting clinical treatments and interventions [20-22], and assisting in mental health counseling and education [17,20,23,24]. Nonetheless, few comprehensive reviews have yet synthesized these applications, assessed the performance of LLMs, or elucidated their advantages within the mental health domain [25,26]. Therefore, we conducted this scoping review to address 4 questions. First, we identified the challenges in mental health and compared the processes adopted by humans, nontransformer models, and LLMs. Second, we summarized the main areas of LLMs’ applications and presented specific processes of these applications. Third, we examined comparative performance studies between LLMs and humans, as well as among different LLMs. Finally, we presented the fine-tuning LLMs for mental health and compared their advantages and disadvantages, which could be directly used by researchers and psychiatrists. This review aimed to provide a foundational understanding of LLM applications in mental health by examining current trends, comparing performance, identifying challenges, and outlining a road map for future research and clinical practice.

## Methods

### Protocol Registration

We drafted the study protocol based on the relevant items from the PRISMA-ScR (Preferred Reporting Items for Systematic Reviews and Meta-Analyses Extension for Scoping Reviews; Multimedia Appendix 1). Compared with the original protocol, the previous query strings were capable of searching for articles related to the applications of LLMs in mental health. However, to enhance the reproducibility of the search process, we have added more detailed query strings. As for databases used, we excluded the Google Scholar database from our search strategy for two reasons: (1) the other 4 English language databases we selected already included the relevant articles and (2) it is not feasible to accurately count the specific number of articles retrieved from the Google Scholar database. Instead, we used official academic databases for the article search. These databases have strict selection criteria and typically include only peer-reviewed, high-quality publications from reputable publishers. In addition, we have updated the literature search period, revising the range from January 1, 2017, to August 31, 2024. This change is important for the following reasons. First, at the beginning of the study, we searched for articles published between January 1, 2017, and August 31, 2024. However, we found that all articles meeting the inclusion criteria were published after 2019. Second, previous studies [9,25] suggested that the term “LLM” was first proposed and widely used starting in October 2019. Therefore, we have revised our inclusion criteria to include only studies published from January 1, 2019, to August 31, 2024. The final protocol was registered prospectively in the Open Science Framework [27].

### Search Strategy and Selection Criteria

A scoping review is a preliminary systematic review that aims to map the existing evidence on a specific topic or field of research [28]. It provides a broad overview of the literature by identifying the nature and extent of existing research, including types of studies, variables, and gaps in the evidence base [29]. This approach is particularly useful when the body of evidence is large or diverse, or when there is a need to understand the scope of a research area before conducting a more focused systematic review.

This scoping review followed the five-stage framework: (1) identifying the research question; (2) identifying relevant studies; (3) study selection; (4) charting the data; and (5) collating, summarizing, and reporting the results. The search terms for mental health include the following: “psychiatr*,” “mental health,” “depress*,” “anxiety,” “posttraumatic stress disorder,” “PTSD,” “bipolar disorder,” “schizophrenia,” “obsessive-compulsive disorder,” “personality disorder,” “insomnia,” and “suicid*.” The keywords and search terms for LLMs in mental health include the following: “large language model,” “OpenAI language model,” “generative AI,” “generative artificial intelligence,” “BERT,” and “GPT.”

According to previous studies [9,25], the term “LLM” is used to distinguish language models based on their parameter scale (eg, containing tens or hundreds of billions of parameters). The term “LLM” has been proposed and widely used since October 2019. In addition, we planned to conduct this scoping review on August 31, 2024. Thus, we searched 4 English language databases (Web of Science, PubMed, IEEE Xplore, and Cochrane Library) and 3 Chinese language databases (Weipu, CNKI, and Wanfang) for peer-reviewed articles published between January 1, 2019, and August 31, 2024. We only included papers in Chinese published in high-quality journals. To find other possibly relevant studies and reports that were missed by the automated searches, the reference lists of the included articles and reports were examined.

The inclusion and exclusion criteria for studies are shown in Textbox 1.

**Study inclusion and exclusion criteria.**

**Inclusion criteria**

- Studies focused on the applications of large language models (LLMs) in the mental health field.
- The LLMs included, but were not limited to, GPT-3/4, ChatGPT, Pathways Language Model (PaLM), LLaMA, and the improved and fine-tuned LLMs.
- Published in a peer-reviewed journal or conference.

**Exclusion criteria**

- Articles that were not peer-reviewed or did not focus on mental health or mental disorders.
- Studies that did not use large language models (those using natural language processing or long short-term memory were also excluded).

The first round of screening was based on titles, keywords, and abstracts (4392/4859, 90.39%). These articles were divided into 6 subsets (each subset includes 732 papers). A total of 6 researchers (JL, PL, BW, YY, HZ, and CN) independently reviewed each part of the papers, including the titles, keywords, and abstracts. During the initial review, each article was categorized into one of the following groups: (1) fully met the inclusion criteria, (2) did not focus on mental health or mental disorders, (3) did not use LLMs, and (4) had unclear eligibility for inclusion. In the second round of screening, we conducted a cross-check process to ensure the classification of these articles (eg, CN reviewed the paper part handled by HZ, and HZ reviewed the paper part handled by JL). Articles from the fourth group were discussed by YJ and YW one by one for their eligibility. Several questions were discussed:

- NLP use uncertainty—some articles used NLP, but it was unclear whether LLMs were used. A full-text review was necessary to make a definitive determination. Thus, we reserved these articles for further review.
- Exclusion of cognitive disorders—it remains uncertain whether aphasia should be classified as part of the mental health domain. After the discussion, we excluded the articles about aphasia as they were not considered within the scope of mental health.
- Electronic medical records (EMRs) in the context—some articles mentioned EMRs, which could potentially be relevant to the mental health domain. However, these articles were not specifically focused on mental health. After the discussion, we decided to reserve these articles for further review.

The third round of review was based on the full texts to assess their eligibility (308/4392, 7.01%). These potentially eligible articles were divided into 6 parts (each part included 51 or 52 papers). A total of 6 researchers (JL, PL, BW, YY, HZ, and CN) independently reviewed each part of the papers, including the titles, abstracts, and full texts of each paper. To ensure the accuracy of paper screening, we conducted a double-check process. The researchers cross-checked each other’s work, reviewing the paper selection process (eg, JL reviewed the paper part handled by PL, and PL reviewed the paper part handled by BW). Disagreements were discussed with third reviewers (YJ and YW) until a consensus was reached. We excluded preprints, reviews, books, studies that did not use LLMs, and those not published in journals or conferences. Ultimately, 95 articles were retained for the final analysis. The search terms for English language databases and Chinese language databases are shown in Multimedia Appendices 2 and 3.

### Data Extraction, Categorization, and Labeling

The final data collection form used for peer-reviewed articles is shown in Table 1 (N=95). The information of each study included categories, regions, application tasks, mental conditions, data sources, sample information, and applied models. These articles were divided into 6 parts (each part included 15 or 16 papers). A total of 6 researchers (JL, PL, BW, YY, HZ, and CN) independently extracted data from each part of the papers. To ensure the accuracy of data extraction, we conducted a double-check process. The researchers cross-checked each other’s work, reviewing the data extraction process. The excluded studies, along with the reasons for their exclusion (eg, “It’s a preprint”), are listed in Multimedia Appendix 4.

For this scoping review, we developed a categorization framework based on the applications of LLMs in mental health: (1) screening or detection of mental disorders, (2) supporting clinical treatments and interventions, and (3) assisting in mental health counseling and education. At least 1 reviewer categorized each study manually by examining the title and abstract to assign categories. When the study categories could not be clearly determined, the methods and results sections were reviewed to assist with classification.

**Table 1:** The basic information of the included studies (N=95).

Categories and study			Basic information							Data information					Models
			Region		Application		Mental conditions		Data sources			Sample information			
**Depression detection and classification**															
	[20]	Israel		Prognosis		Depression		Case vignettes and previous studies			1074 experts		GPT-3.5, GPT-4, and Claude and Bard		
	[24]	United States		Prediction		Stress and depression		Dreaddit, CSSRS-Suicide			3553 posts		Mental-Alpaca and Mental-FLAN-T5		
	[30]	Singapore		Analysis		Depression, PTSD^a^, anxiety		Reddit, SMS text messaging, Twitter, and MHI dataset			105,000 data samples		MentaLLaMA		
	[31]	United States		Detection		Depression, anxiety, SI^b^		Reddit and Twitter			Conversations		PsychBERT		
	[32]	United States		Detection		Depression		Twitter			2575 tweets from Twitter users with depression		BERT^c^, RoBERTa^d^, and XLNet		
	[33]	India		Detection		Depression		Twitter			189 interviews		BERT with multimodal frameworks		
	[34]	China		Detection		Depression		DAIC-WOZ^e^			Respondents with depression labels		BERT		
	[35]	United Arab Emirates		Detection		Depression		E-DAIC^f^			7650 unique entries		BERT-based custom classifier		
	[36]	Canada		Prediction		Depression, ADHD^g^, anxiety		Reddit			2514 users’ posts and 167,444 clinical posts, 2,987,780		BERT, ROBERTa, open AI GPT, and GPT 2		
	[37]	China		Detection		Depression and SI		Dialogues from real-life scenarios			Depression (64 samples) and anxiety (75 samples)		GPT-3.5		
	[38]	United States		Detection		Depression		DAIC^h^, E-DAIC, and EATD			Nondepression and depression, DAIC, E-DAIC, and EATD		BERT and RoBERTa		
	[39]	China		Detection		Depression		DAIC-WOZ			189 participants		BERT		
	[40]	United States		Detection		Depression		Twitter (sentiment dataset)			632,000 tweets		RoBERTa, DeBERTa, DistilBERT, and SqueezeBERT		
	[41]	Malaysia		Detection		Depression		Interviews, Facebook, Reddit, and Twitter			53 participants (11 of them were with depression)		GPT-3 (ADA model)		
	[42]	United States		Detection		Depression		DAIC-WOZ, extended-DAIC, and simulated data			DAIC-WOZ and E-DAIC (data from 122 participants with depression)		BERT, GPT-3.5, and GPT-4		
	[43]	China		Prediction		Depression		Weibo			13,993 microblogs with depression labels		BERT, RoBERTa, and XLNET		
	[44]	United Kingdom		Detection		Depression		RSDD and RSDD-Time			Posts (9210 users with depression)		ALBERT, BioBERT, Longformer, MentalBERT, and MentalRoBERTa		
	[45]	Switzerland		Classification		Depression		DAIC-WOZ			Respondents with depression labels		BERT, RoBERTa, and DistilBERT		
	[46]	United States		Responses		PPD^i^		ACOG^j^ and PPD			14 questions		GPT-4 and LaMDA		
	[47]	Germany		Detection		Depression		E-DAIC			275 participants		DepRoBERTa		
	[48]	Canada		Detection		Depression		DTR dataset			42,691 tweets from Depression-Candidate-Tweets, 6077 tweets from Depression Tweets Repository and 1500 tweets from DSD-Clinician-Tweets		Mental-BERT		
	[49]	Malaysia		Detection		Depression		Scraped and survey PHQ-9^k^			250 users		BERT		
	[50]	Netherlands		Detection		Depression		Clinical data (16,159 patients)			Survey PHQ-9 and scraped		DistilBERT		
	[51]	Greece		Detection		Stress and depression		Dreaddit dataset			16,159 patients		M-BERT and M-MentalBERT		
	[52]	Israel		Evaluations		Depression		Clinical vignettes			14 questions		GPT-3.5 and GPT-4		
	[53]	United States		Screening		Depression		DAIC-WOZ			Diagnosed with depression or 8 vignettes		AudiBERT (I, II, and III)		
	[54]	Canada		Assessment		Depression		DAIC-WOZ			15 thematic datasets		Prefix-tuned RoBERTa		
	[55]	India		Detection		Depression		Reddit (mental health corpus and depression)			189 participants		RoBERTa		
	[56]	Iran		Prediction		Depression		Autodep dataset (Twitter)			219 samples and 20 real participants		DBUFS2E, BBU, MBBU, and DRB		
	[57]	United States		Classification		Depression		GLOBEM dataset			Collection of passive sensing or tweets and bioscriptions		GPT-3.5, GPT-4 and pathways language model 2		
	[58]	Russia		Detection		Depression		DAIC-WOZ			Respondents with depression labels		BERT, MentalBERT, MentalRoBERTa, PsycBERT, and ClinicalBERT		
	[59]	China		Diagnosis		Depression		Labeled text data			NR^l^		DepGPT		
	[60]	South Korea		Detection		Depression		Mind station app data			428 diaries		GPT-3.5 and GPT-4		
**Suicide**															
	[17]	United States		Prediction		SI		Brightside Telehealth platform			460 (SI at intake, SI later, and without SI)		GPT-4		
	[23]	Austria		Detection		Suicide		Twitter			3202 English tweets		BERT and XLNet		
	[24]	United States		Prediction		Stress and depression		Dreaddit, CSSRS-Suicide			3553 posts		Mental-Alpaca and Mental-FLAN-T5		
	[30]	Singapore		Analysis		Depression, PTSD^a^, anxiety		Reddit, SMS, Twitter, and MHI dataset			105,000 data samples		MentaLLaMA		
	[31]	United States		Detection		Depression and anxiety		Reddit and Twitter			148,700 conversations		PsychBERT		
	[61]	Morocco		Detection		Suicide		Reddit			Suicide and nonsuicide content or 232,074 posts		GPT and BERT		
	[62]	Canada		Classification		Suicide		Reachout.com forum posts and UMD Reddit dataset			Posts in/r/SuicideWatch on Reddit or 1588 labeled posts		GPT-1		
	[63]	Israel		Assessment		Suicide		Professional mental health assessments			4 vignettes		GPT-3.5 and GPT-4		
	[64]	Canada		Detection		SI		UMD dataset and LLM^m^ synthetic datasets			>100,000 posts and comments		BERT		
	[65]	Brazil		Detection		SI^b^		Twitter			Suicide-related text or 5699 tweets		Boamente		
	[66]	China		Classification		Suicide		Microblog user data (ZouFan comments)			4500 pieces		Knowledge-perception BERT model		
	[67]	United States		Detection		Suicide		Reddit (SuicideWatch section)			2.9 million posts and 30 sub-Reddits (including mental health and control sub-Reddits)		BERT		
**Other mental disorders**															
	[24]	United States		Prediction		Stress and depression		Dreaddit, CSSRS-Suicide			3553 posts		Mental-Alpaca and Mental-FLAN-T5		
	[30]	Singapore		Analysis		Depression, PTSD^a^, anxiety		Reddit, SMS, Twitter, and MHI dataset			105,000 data samples		MentaLLaMA		
	[31]	United States		Detection		Depression and anxiety		Reddit and Twitter			148,700 conversations		PsychBERT		
	[68]	India		Detection		Stress and anxiety		Reddit			3553 labeled posts		RoBERTa and XLNet		
	[69]	India		Detection		Stress, depression, and suicide		Reddit and Twitter			Stress, depression, and suicide		GPT-2 and GPT-Neo-125M		
	[70]	Canada		Screening		GAD^n^		Prolific platform data			2000 participants		Longformer		
	[71]	United States		Diagnosis		OCD^o^		Clinical vignettes			OCD vignettes		GPT-4, Gemini pro, and LLaMA 3		
	[72]	Iran		Diagnosis		Mental health disease		Clinical cases			Selected by a medical expert or 20 cases		GPT-3.5, GPT-4, Nemotron, and Aya		
	[73]	China		Prediction		Mental disorder		Kaggle			16,950 categories or 41,851 reviews		MentalBERT		
	[51]	Greece		Detection		Stress and depression		Dreaddit dataset			16,159 patients		M-BERT and M-MentalBERT		
	[74]	Israel		Diagnosis		BPD^p^ and SPD^q^		Emotional scenarios (20 cases)			20 scenarios		GPT-3.5		
	[75]	United States		Rating		Emotion		Psychotherapy transcripts and interrater dataset			97,497 ratings		BERT		
	[76]	China		Extraction		Psychiatric disorder		Clinical notes (12,006 records)			Human or 12,006 anonymous clinical notes		BERT		
	[77]	China		Screening		Schizophrenia, BPD, major depressive disorder, and DD		EHRs^r^			500 EHRs		BERT, RoBERTa, DistilBERT, and ALBERT		
	[78]	Iran		Diagnosis		Depression, OCD, GAD, BPD, and schizophrenia		*Diagnostic and Statistical Manual of Mental Disorders, Fifth Edition*–based case scenarios			13 case scenarios		GPT-3.5, GPT-4, AYA, and Nemotron-3-8B		
	[79]	United States		Text analysis		Sentiment		Tweets and news			Annotated by human or 47,925 tweets and news headlines		GPT3.5 turbo, GPT4, and GPT4 turbo		
	[80]	United States		Sentiment		Sentiment		Multiple sources			Tokens		OPT, GPT-3.5, and BERT		
	[81]	United States		Classification		Emotion and sentiment		Social media			417,423 and 1,176,509 samples		EmoBERTTiny		
	[82]	United States		Emotion		Depression		Stress and coping process questionnaire			100 nonstudent adults		Text-davinci-003, GPT-3.5, and GPT-4		
	[83]	India		Identification		Emotion		GoEmotions dataset and Twitter dataset			27 different emotion categories or comments and tweets		MobileBERT		
	[84]	Israel		Emotion		Emotion		RMET and LEAS			36 photos and 20 questions		GPT-4 and Bard		
	[85]	United States		Classification		Psychotherapy		Smart home images			7 different environments or 10,767 images		GPT-4		
**Supporting clinical treatments**															
	[15]	Canada		Diagnosis		PTSD		E-DAIC dataset and ChatGPT-generated transcripts			Severe depression and PTSD or 219 participants		GPT-3.5-turbo		
	[16]	United States		Detection		CB-PTSD^s^		Participant narratives			1295 narratives		GPT-3.5-turbo		
	[18]	South Korea		Therapy		ADHD and dementia		USPTO patent data			8656 patients and 205 DTx patents		BERTopic and PatentSBERTa		
	[20]	Israel		Assessment		Depression		Case vignettes and previous studies			1074 experts		GPT-3.5, GPT-4, Claude, and Bard		
	[21]	United States		Disorder		Bipolar depression		EHR-based generated data			50 sets of clinical vignettes		GPT-4		
	[86]	United States		Screening		General mental health issues		EHRs and clinical notes			2,476,628 patients or 290,482,002 clinical notes		GatorTron		
	[87]	China		Counseling		Stress, LGBTQ issues		Consultation web sites, Weibo, and Zhihu			31 unique questions		ChatGLM, ERNIE Bota, and Qianwen		
	[88]	United States		Counseling		NR		MentalCLOUDS dataset			11,543 utterances		BART, T5, GPT series, Phi-2, MentalBART, Flan-T5, Mistral, LLaMA-2, and MentalLLaMA		
	[89]	United Kingdom		Therapy		Anxiety		Therapist-written thoughts			20 tasks at each of 3 stages		GPT-4 and Bard		
	[90]	United Kingdom		Questionnaires validation		Depression, anxiety, and PTSD		C19PRC study data			2058 adults		Sentence-BERT		
	[91]	Australia		Diagnosis		Various psychiatric conditions		Clinical case vignettes			100 cases		GPT-3.5		
	[92]	China		Diagnosis		Depression		MedDialog, Metal Real			NR		LLaMA, ChatGLM, and Alpaca		
	[93]	United States		Analysis		Psychoactive experiences		Erowid and PDSP-Ki dataset			11,816 testimonials		BERTowid, BERTiment, and CCA		
	[94]	India		Prediction		Stress and anxiety		Survey dataset			41,000 entries		Gemini		
**Chatbots**															
	[20]	Israel		Assessment		Depression		Case vignettes and previous studies			2 vignettes differed in gender		GPT-3.5, GPT-4, Claude, and Bard		
	[22]	China		Chatbots		Autistic		DXY platform (medical consultation data)			100 consultation samples		GPT-4 and ERNIE bot		
	[35]	United Arab Emirates		Detection		Depression		E-DAIC dataset			219 samples and 20 real participants or 219 E-DAIC samples		BERT-based custom classifier		
	[95]	United States		Evaluation		Depression and SI		Human made			25 conversational agents		GPT-3.5		
	[96]	Spain		Emotion		General emotional states		Internet-based human conversations			64 participants		GPT-3		
	[97]	Germany		Therapy		ADHD		NR			NR		GPT3.5, GPT-4 turbo, and Claude-3 opus		
	[98]	Poland		Sentiment		Mental health		Corpus of Translated Emotional Texts and Polish common crawl			Sentences and web pages		GPT-3.5		
	[99]	United States		Detection		Loneliness and SI		Survey data			1006 users of Replika		Replika		
**Data augmentation**															
	[15]	Canada		Diagnosis		PTSD		E-DAIC dataset and ChatGPT-generated transcripts			Severe depression and PTSD or augmented data		GPT-3.5		
	[16]	United States		Detection		CB-PTSD		Participant narratives			1295 narratives		GPT-3.5-turbo		
	[47]	Germany		Detection		Depression		E-DAIC dataset			275 participants		DepRoBERTa		
	[64]	Canada		Detection		SI		UMD dataset and LLM synthetic datasets			>100,000 posts and comments		BERT		
	[92]	China		Diagnosis		Depression		EATD-Corpus, MedDialog dataset (Chinese)			NR		LLaMA-7B, ChatGLM-6B, and Alpaca		
	[100]	Canada		Augmentation		PTSD		E-DAIC dataset, generated data			219 interview records		CALLM^t^, GPT-4, DistilBERT, and BERT		
	[101]	China		Augmentation		Mental health		ChatGPT-generated narratives			3017 instances; 80/20 train-test split		BERT, BLOOM-560M, BLOOMZ-3B, ChatGLM2-6B, and Baichuan-7B		
	[102]	Turkey		Generation		Various disorders		DAIC-WOZ, ChatGPT-dataset, and Bard-dataset			Real patients and synthetic patients		GPT-3.5 and Bard		
	[103]	China		Generation		Psychiatry		DSM-5 diagnostic criteria			2000 records		Mistral7B-DSM-5 model		
**Assisting in counseling**															
	[104]	India		Chatbot		Depression and anxiety		Reddit			Questions related to the illness or NR		CareBot		
	[105]	United States		Chatbot		General mental health		Reddit			120 posts (2917 user comments)		Replika		
	[106]	Poland		Chatbot		General mental health		Empathetic dialogues and DailyDialog datasets			DailyDialog dataset or NR		BERT		
	[14]	Philippines		Chatbot		Not suitable		Well-being conversations, PERMA Lexica			24,850 conversations		VHope		
	[107]	Canada		Chatbot		General mental well-being		Prompts made by author			With mindfulness experience or NR		GPT-3 based chatbots		
	[108]	United States		Health care		General mental well-being		NR			NR		GPT-4o		
	[99]	United States		Detection		Loneliness and suicide		Survey data			1006 users of Replika		Replika		
	[109]	United States		Generation		Depression		Psychiatric questions			4 questions		GPT-3.5 and GPT4		
	[110]	United Kingdom		Measurement		General mental health		Qwell platform therapy transcripts			254 conversations		RoBERTa and CTM^u^		
**Assisting in mental health** **education**															
	[19]	United States		Education		ADHD and ED^v^		Interview data			With signs of a disorder or 102 students		GPT-3		
	[111]	Australia		Education		Substance use		Mental health portals			“Cracks in the Ice” website		GPT-4		

^a^PTSD: posttraumatic stress disorder.

^b^SI: suicidal ideation.

^c^BERT: bidirectional encoder representations from transformers.

^d^RoBERTa: a robustly optimized bidirectional encoder representations from transformers pretraining approach.

^e^DAIC-WOZ: Distress Analysis Interview Corpus-Wizard of Oz.

^f^E-DAIC: Extended Distress Analysis Interview Corpus.

^g^ADHD: attention-deficit hyperactivity disorder.

^h^DAIC: Distress Analysis Interview Corpus.

^i^PPD: postpartum depression.

^j^ACOG: American College of Obstetricians and Gynecologists.

^k^PHQ: Patient Health Questionnaire

^l^NR: not reported.

^m^LLM: large language model.

^n^GAD: generalized anxiety disorder.

^o^OCD: obsessive-compulsive disorder.

^p^BPD: borderline personality disorder.

^q^SPD: schizoid personality disorder.

^r^EHR: electronic health record.

^s^CB-PTSD: childbirth related posttraumatic stress disorder.

^t^CALLM: Clinical Interview Data Augmentation via Large Language Models.

^u^CTM: contextualized topic model.

^v^ED: erectile dysfunction.

### Statistical Analysis

Descriptive statistics were used to summarize the distribution of studies across different application areas. For each application task, we calculated frequencies and percentages. For the performance comparisons between humans and LLMs, between LLMs and nontransformer models, also between various LLMs, we collected the metrics results and plotted the results. Calculations and data charting were performed using R software (version 4.4.2) developed at Bell Laboratories by John Chambers and colleagues, and PyCharm software developed by JetBrains.

## Results

### Overview

**Figure 1:**
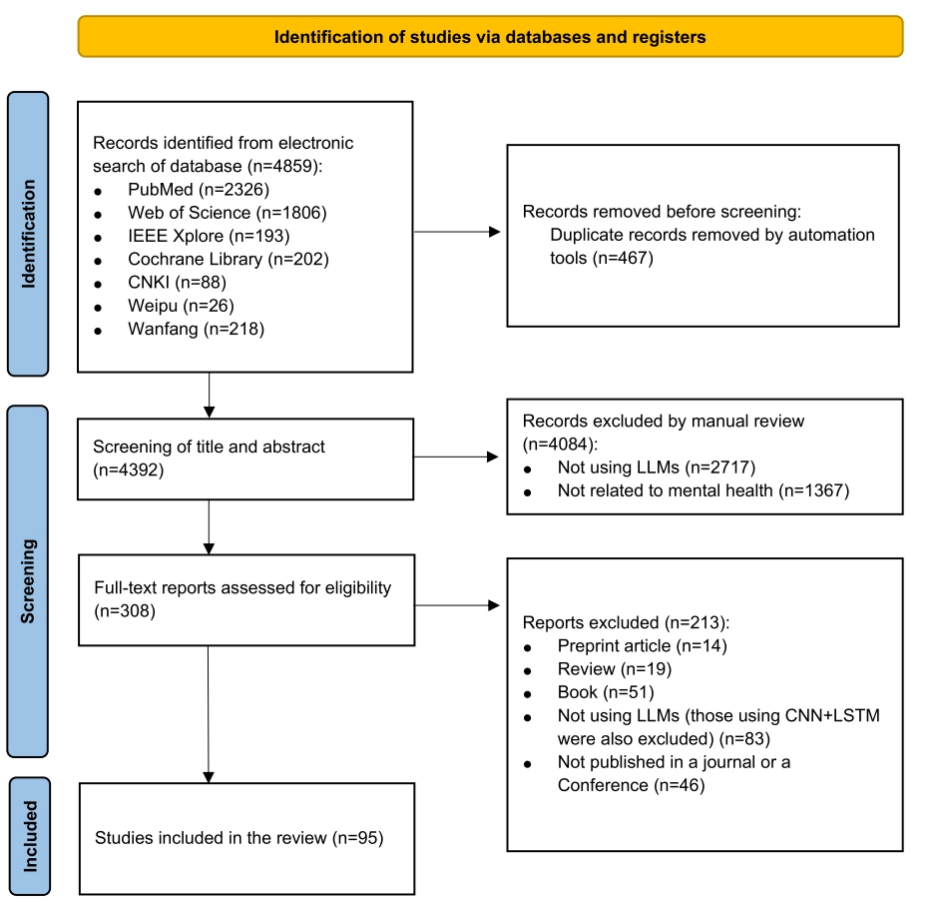
PRISMA (Preferred Reporting Items for Systematic reviews and Meta-Analyses) flowchart of the studies identified for inclusion in the scoping review about applications of large language models (LLMs) in mental health. CNN: convolutional neural networks; LSTM: long short-term memory.

The initial search yielded 4859 records, of which 467 duplicates were removed. Of the remaining 4392 records, 4084 records were removed due to the contents irrelevant to mental health or not using LLMs. After the full-text screening, 95 articles fulfilled the inclusion criteria (Figure 1). Table 1 demonstrates the basic information of each study, including categories, regions, application tasks, mental conditions, data sources, sample information, and applied models.

### Comparisons Between LLMs, Nontransformer Models, and Humans

**Figure 2:**
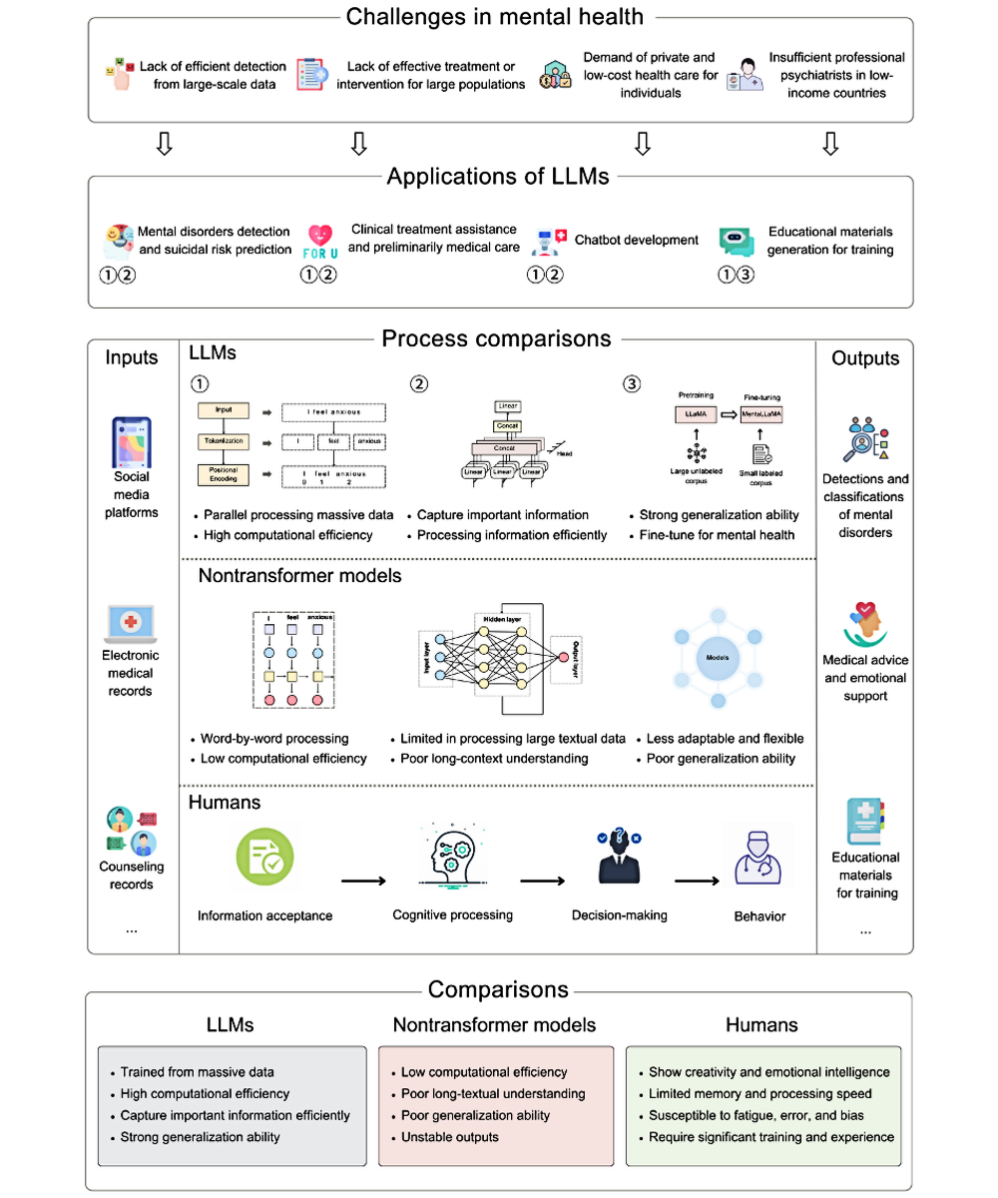
The current challenges of the mental health field; comparisons between large language models (LLMs), nontransformer models, and humans. LLaMA: large language model; Meta AI; Mental-LLM: large language model for mental health.

The current challenges in the mental health field include difficulty in efficient mental disorders detection from large-scale data, effective treatment or intervention for large populations, private and low-cost health care, demand for professional psychiatrists, and so on. Compared with nontransformer models and humans, LLMs present higher capabilities in efficient parallel computing of massive data, textual generation, information capture, strong generalization ability, and fine-tuned mental health tasks. The process comparisons between LLMs, nontransformer models, and humans were presented with the framework (Figure 2, a higher resolution version of figure is also available in Multimedia Appendix 5). Therefore, LLMs have been applied to tasks such as detecting or predicting mental disorders, supporting clinical treatment, providing preliminary medical care, and generating educational materials—using datasets from sources such as social media platforms, EMRs, and counseling transcripts.

### Categorization of Studies Based on Mental Health Applications

We categorized studies in terms of the applications of LLMs in the mental health field. These applications were divided into 3 categories: screening or detection of mental disorders (67/95, 71%), supporting the clinical treatments and intervention (31/95, 33%), and assisting in mental health counseling and education (11/95, 12%; Multimedia Appendix 6). Each study was categorized with ≥1 applications; thus, the percentages sum to >100%. Most studies applied LLMs for depression detection and classification (33/95, 34.7%), supporting clinical treatments and interventions (14/95, 15%), and suicide risk prediction (12/95, 13%; Multimedia Appendix 6). These studies used data from social media platforms such as Reddit, Twitter, Facebook, and Weibo, or clinical datasets (Distress Analysis Interview Corpus-Wizard of Oz and Extended Distress Analysis Interview Corpus), as well as semistructured interviews from hospitals. When evaluating the performance of these LLMs, most studies measured the performance of LLMs with various metrics, such as *F*_1_-score (54/95, 57%), precision (34/95, 36%), accuracy (45/95, 47%), and recall (32/95, 34%).

**Figure 3:**
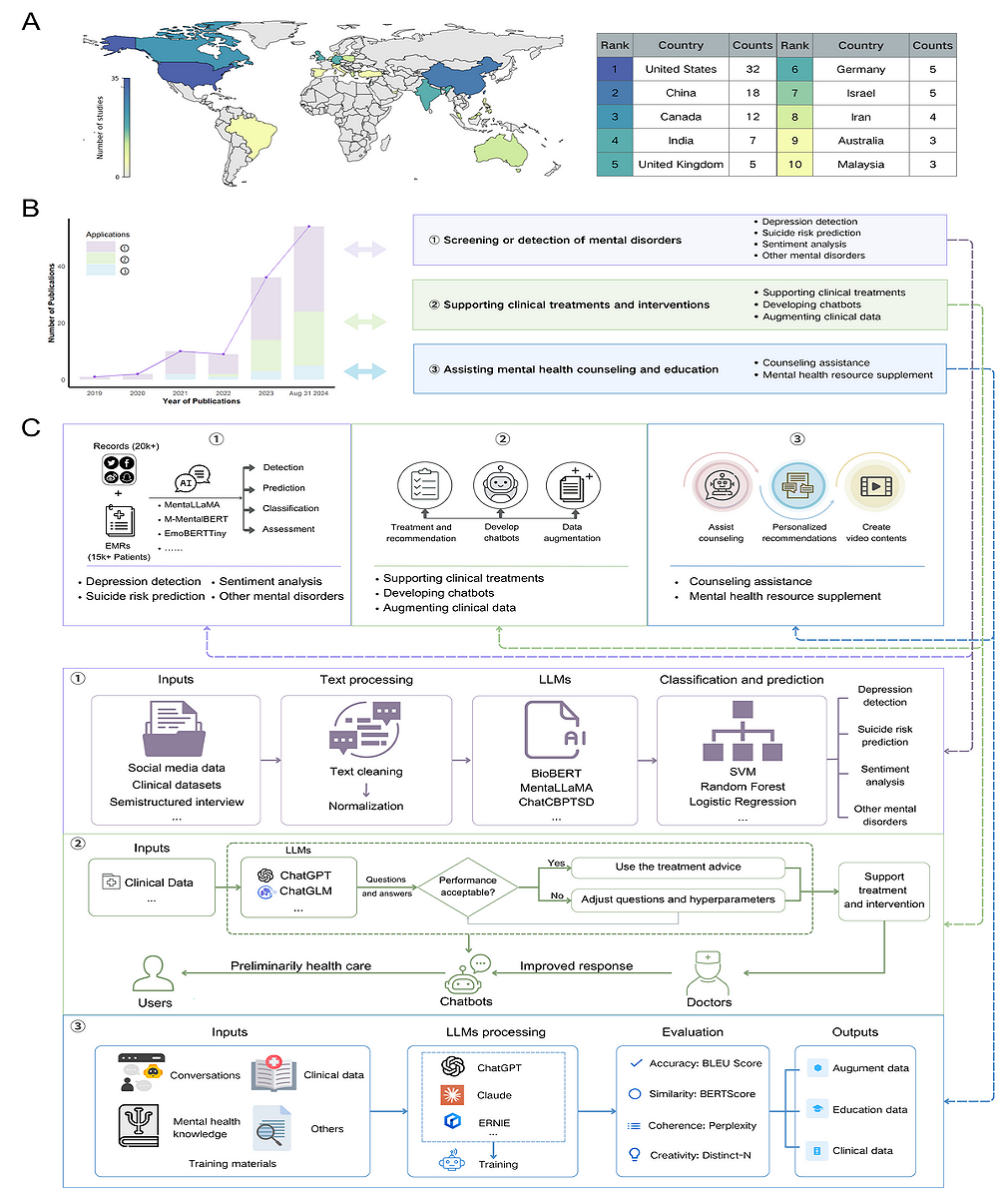
Trends in and applications of large language models (LLMs) in mental health: (A) number of studies mapped by country, (B) trends of included studies published per year, and (C) the framework of 3 application categories of LLMs. BERT: bidirectional encoder representations from transformers; BERTScore: bidirectional encoder representations from transformers–based evaluation score; BioBERT: biomedical bidirectional encoder representations from transformers; BLEU: bilingual evaluation understudy; ChatCBPTSD: chat-based cognitive behavioral therapy for posttraumatic stress disorder; ChatGLM: chat general language model; Distnct-N: automatic metric for evaluating diversity in language generation tasks; EMR: electronic medical record; EmoBERTTiny: emotion-aware bidirectional encoder representations from transformers tiny; ERNIE: enhanced representation through knowledge integration; MentalLLaMA: large language model for mental health; M-MentalBERT: multilingual mental health bidirectional encoder representations from transformers; SVM: support vector machine.

The number of studies mapped by country is presented in Figure 3A (a higher resolution version of figure is also available in Multimedia Appendix 5). The United States is the country that has explored the applications of LLMs in the field of mental health the most, followed by China and Canada. The number of included studies increased year on year, and this trend is shown in Figure 3B. We can find that since 2019, an increasing number of researchers have been exploring the applications of LLMs in the field of mental health. As for the application areas, the first area mainly focused on screening or detection of mental disorders, including depression detection, suicide risk prediction, sentiment analysis, and other mental disorders. The second area focused on supporting clinical treatments and interventions, including supporting clinical treatments, developing chatbots, and augmenting clinical data. The third area focused on assisting mental health counseling and education, including counseling assistance and mental health resource supplement (Figure 3C). These studies applied basic LLMs or fine-tuned LLMs (eg, MentaLLaMA [30], PsychBERT [31], and RoBERTa [68]) to detect or predict depression [30,32-41,49,61,69]; suicide risk [23,24,62]; and other mental disorders, such as anxiety [37,70], obsessive-compulsive disorder [71], and posttraumatic stress disorder [15]. The detailed process for these applications of LLMs is presented in Figure 3C.

In the second application area, most studies explored the capability of LLMs in supporting clinical treatments and interventions [17,21,23,62,63,86], developing chatbots, and augmenting unbalanced clinical data [95,104-106]. These studies applied LLMs to provide treatment advice, assist diagnostic services, and assess prognosis through a question-answering approach. The performance of LLMs was evaluated by professional clinicians and compared with the related performance of humans. A total of 3 studies also applied LLMs to elicit emotion [20-22]. Furthermore, to address the imbalance of clinical data and enhance diagnosis and treatment, LLMs could augment clinical data and targeted dialogues in safe and stable ways.

Moreover, LLMs have been applied for counseling assistance [14,104-106] and educational resource supplements [19,111]. These results showed that the introduction of any interaction (video or chatbot) improved intent to practice and overall experience compared to the baseline [107]. Furthermore, LLMs showed the potential to generate educational materials for training.

### Performance Comparisons Between LLMs and Humans and Between Various LLMs

**Figure 4:**
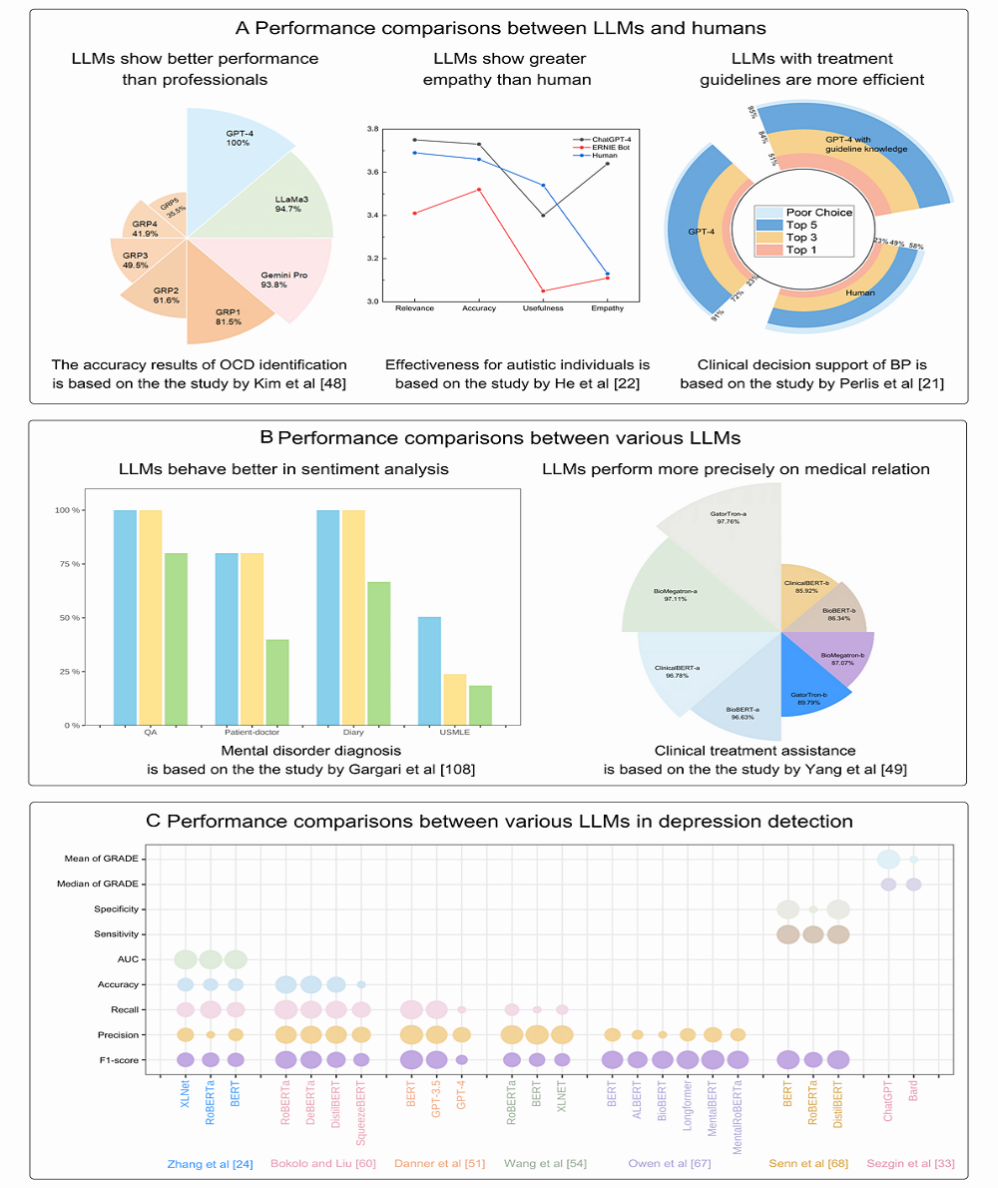
Performance comparisons between large language models (LLMs) and humans and between various LLMs: (A) performance comparisons between LLMs and humans, (B) performance comparisons between various LLMs, (C) performance comparisons between various LLMs in depression detection. AUC: area under the curve; BioBERT: biomedical bidirectional encoder representations from transformers; BP: bipolar depression; ClinicalBERT: clinical bidirectional encoder representation from transformers; ERNIE: enhanced representation through knowledge integration; GPT: generative pretrained transformer; GRADE: Grading of Recommendations Assessment, Development and Evaluation; GRP: group; GRP1: doctoral trainees; GRP2: APA members; GRP3: primary care physicians; GRP4: medical providers; GRP5: clergy members; OCD: obsessive-compulsive disorder; poor choice: poor or contraindicated medications; QA: question and answers; Top 1/3/5: the first 1/3/5 plans with optimal decisions; USMLE: United States Medical Licensing Exam.

Several studies compared the performance between humans and LLMs. The metrics’ results of performance were displayed in Multimedia Appendix 7. Figure 4A (a higher resolution version of figure is also available in Multimedia Appendix 5) presents the results of 3 studies on obsessive-compulsive disorder identification [71], chatbots efficiency [22], and treatment support [21]. According to these results, most LLMs showed efficient and promising performance for mental health tasks. Several LLMs, such as GPT-4, Claude, and Bard, aligned closely with mental health professionals’ perspectives.

Figures 4B and 4C show the comparisons of model performance between different LLMs in mental disorder diagnosis [72], clinical treatment assistance [86], and depression detection [32,40,42-46]. These results found that the latest LLMs (eg, ChatGPT) perform better than traditional and previous models. The complete results are presented in Multimedia Appendix 8.

### Existing Fine-Tuned LLMs for Mental Health

Table 2 provides the fine-tuned LLMs for mental health, including availability, base models, the number of parameters, training strategy, and published year. These fine-tuned LLMs could be applied specifically to mental health tasks.

**Table 2:** Fine-tuned large language models (LLM) for mental health.

Availability of the fine-tuned models and base models			Fine-tuned models		Parameters		Training strategy		Year
**Yes**									
	BERT^a^	MBBU^b^		Unreported		Fine-tuning		2024	
	BERT	BioBERT		Unreported		Unreported		2023	
	BERT	MentalRoBERTa		Unreported		Unreported		2023	
	BERT	PsychBERT		Unreported		Domain adaptation (domain-adaptive pretraining)		2021	
	LLaMA	MentaLLaMA		7 billion-13 billion		IFT^c^		2024	
	LLaMA	ChatCounselor		7 billion		IFT		2023	
	FLAN-T5^d^	Mental-FLAN-T5		7 billion-1700 billion		IFT		2024	
	FLAN-T5	Mental-LLM		7 billion or 11 billion		IFT		2023	
	GPT	LLM-counselors		Unreported		TFP^e^		2024	
	GPT	ChatCBPTSD		Unreported		TFP		2023	
	Alpaca	Mental-alpaca		7 billion-1700 billion		IFT		2024	
**Not specified**									
	BERT	CALLM^f^		Unreported		IFT		2024	
	BERT	EmoBERTTiny		4.4 million		IFT		2024	
	BERT	M-MentalBERT		Unreported		IFT		2024	
	BERT	Boamente		Unreported		IFT		2022	
	BERT	AudiBERT (I, II, and III)		Unreported		IFT		2021	
	GPT	Psy-LLM		Unreported		TFP		2023	
	GPT	CareBot		Unreported		IFT		2021	

^a^BERT: bidirectional encoder representation from transformers.

^b^MBBU: mentalBERT-base-uncased.

^c^IFT: instruction fine-tuning.

^d^FLAN-T5: fine-tuned language net-t5.

^e^TFP: tuning-free prompting.

^f^CALLM: a framework for systematic contrastive analysis of large language models.

### Common Advantages and Disadvantages

**Figure 5:**
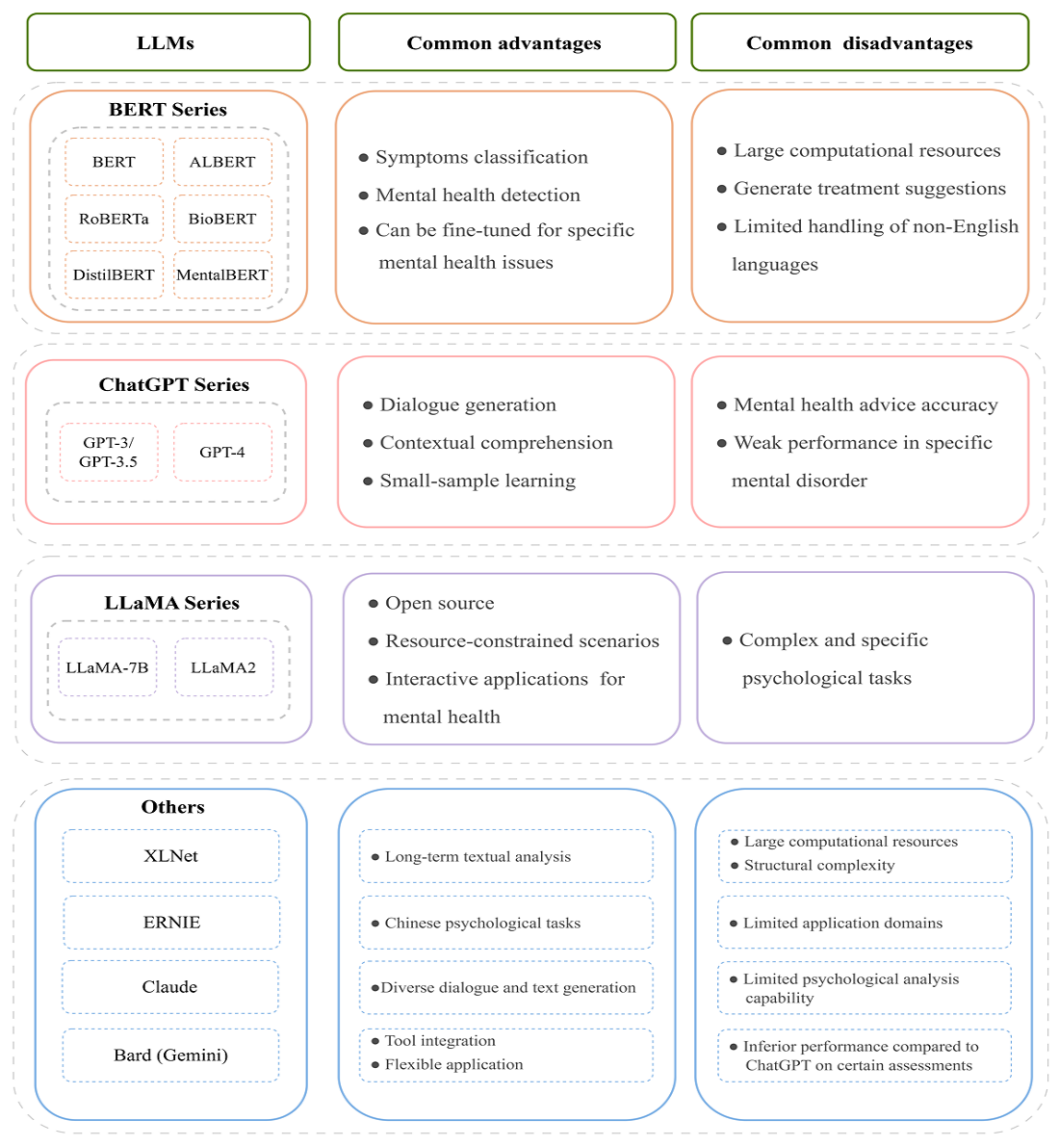
The common advantages and disadvantages of large language models (LLMs). ALBERT: a lite bidirectional encoder representations from transformers; BERT: bidirectional encoder representations from transformers; BioBERT: biomedical bidirectional encoder representations from transformers; DistilBERT: distilled version of bidirectional encoder representations from transformers; ERNIE: enhanced representation through knowledge integration; GPT: generative pretrained transformer; LLaMA-7B: LLaMAwith 7 billion parameters; MentalBERT: bidirectional encoder representations from transformers for mental health; RoBERTa: a robustly optimized bidirectional encoder representations from transformers pretraining approach; XLNet: a unsupervised language representation learning method.

Figure 5 summarizes the common advantages and disadvantages of LLMs in mental health. These LLMs could be divided into the BERT series, GPT series, LLaMA series, and others. For example, the shared strengths of BERT-based models make them well-suited for fine-tuning on specific mental health issues. However, the BERT series models require large computational resources. The ChatGPT series models can conduct multiround dialogue, even based on small-sample learning. Nevertheless, the accuracy of ChatGPT models should be improved. Furthermore, GPT-4 could receive multimodal data and show more powerful performance in comprehension and generation. As for the LLaMA series models, they are open source for the public and beneficial for interactive applications, although their complex task performance is inferior to large-scale proprietary models.

## Discussion

### Principal Findings

This scoping review explored the applications of LLMs in the mental health field and summarized trends, application areas, performance comparisons, challenges, and prospective future directions. The applications of LLMs were categorized into 3 key areas: screening or detection of mental disorders, supporting clinical treatments and interventions, and assisting in mental health counseling and education. Most studies used LLMs for depression detection and classification (33/95, 35%), clinical treatment support and intervention (14/95, 15%), and suicide risk prediction (12/95, 13%). Compared with nontransformer models and humans, LLMs demonstrate higher capabilities in information acquisition and analysis and efficiently generating natural language responses. Furthermore, we summarized the fine-tuning LLMs for mental health and compared their advantages and disadvantages, offering insights for future researchers and psychiatrists.

### Advantages of LLMs’ Applications in Mental Health

Compared to nontransformer models and humans, LLMs demonstrate higher capabilities in information acquisition, analysis, and generating professional responses. These enhanced capabilities posit LLMs as potential tools for the detection and prediction of mental disorders through the analysis of extensive datasets, including social media content [30,32,33], EMRs [21,86], and counseling notes [87,88]. Their applications in treatment and intervention are noteworthy. LLMs could assimilate patient clinical records, summarize treatment sessions, and support diagnoses for mental disorders. This potential streamlines the workflow for patients and mental health care systems efficiently. On the basis of the information interaction and generation ability, LLMs can be instrumental in the development of chatbots designed for initial medical consultation and emotional support. Such applications help to offer discreet and affordable health care solutions to individuals who, due to stigma or financial constraints, are reluctant to seek assistance for mental health issues.

In this scoping review, most studies applied LLMs in detecting depression and suicidal risk [34-41,49,61,69]. These studies have demonstrated the potential of LLMs such as MentaLLaMA [30], PsychBERT [31], RoBERTa [68], and GPT-4 [89], in detecting and identifying mental disorders from social media platforms and clinical datasets. These models have been trained to recognize depression and recommend evidence-based treatments, with some, such as GPT-4 and Claude, closely aligning with the perspectives of mental health professionals [20]. Furthermore, the integration of linguistic features and the appending of mental disorders’ background information have been shown to enhance classification performance and calibration of these LLMs [17,23,62,63]. LLMs perform comparably to experienced clinicians in identifying the risk of suicide ideation, with the addition of suicide attempt history enhancing sensitivity. GPT-4 has demonstrated superior performance over GPT-3.5 in recognizing SI, despite a tendency to underestimate resilience [24]. Other studies showed that the use of LLMs produced effective strategies for predicting suicidal risk with sparsely labeled data [23,62]. This efficiency in labeling and analysis of large datasets is a better advancement over previous methods, which were often hindered by the requirement of manual annotation and consequently limited by small sample sizes or finite datasets.

Due to their efficient ability to acquire information and generate humanlike language, LLMs show potential in preliminary care [14], providing treatment guidelines, augmenting unbalanced clinical datasets [95,104-106], and assisting in training professionals. LLMs also have shown potential to assist with cognitive behavioral therapy tasks, such as reframing unhelpful thoughts and identifying cognitive biases [89]. Several studies also investigated the potential of LLMs in addressing health care regional disparities and enhancing diagnostic and therapeutic services, although these LLMs have only shown initial results so far [20-22]. Furthermore, in the diagnosis of posttraumatic stress disorder [15], LLM-augmented datasets have shown improved performance over original datasets, with both zero-shot and few-shot approaches outperforming the original dataset, highlighting the effectiveness of LLMs in enhancing diagnostic accuracy with minimal additional data. According to the results of performance comparisons, LLMs present similar performance as professionals and may even surpass physicians [22]. These results demonstrate the potential of LLMs in clinical assistance and support. However, when evaluating the quality of LLM-generated responses to queries and educational health materials, several studies have indicated that, despite GPT-4’s strong face validity, it remains insufficiently reliable for direct-to-consumer use [111]. Therefore, the outputs by LLMs for educational health require cautious human editing and oversight. These findings underscore the growing importance of LLMs in mental health research and practice, offering new avenues for early detection, risk assessment, and intervention strategies.

### Prompt Engineering Techniques of LLMs

Prompt engineering techniques are becoming increasingly essential across various applications and mental health conditions [109]. By optimizing prompt strategies in a targeted manner, models can more effectively perform tasks such as the preliminary detection of mental disorders, intervention recommendations, and emotional assessments, all while adhering to professional and ethical standards. Previous studies have reported that the use of diverse prompts when applying LLMs can lead to substantial variations in the stability and accuracy of the models’ outputs [112,113]. Notably, without modifying the underlying architecture of the LLMs, adjusting the input prompts alone can significantly influence the quality and relevance of the outputs. This underscores the critical role of prompt design in optimizing model performance. Thus, it is vital to standardize prompts and share them openly in academic and clinical contexts to enhance the robustness and reproducibility of research findings. In mental health dialogue scenarios, prompts must balance professional standards with ethical considerations to prevent the generation of misleading or inappropriate content [114]. Clinicians should be aware that poorly designed prompts may pose potential risks, such as inaccurate treatment suggestions or unsuitable discussion topics. The ongoing refinement and standardization of prompt engineering not only enhance the performance and explainability of LLMs but also enable health care professionals and researchers to provide more efficient and safer preliminary support and services to patients [115].

### Challenges of LLMs’ Applications in Mental Health

While LLMs have demonstrated high performance on certain benchmarks, their practical applications in clinical settings are still limited. Recent studies have shown that even advanced LLMs struggle with complex clinical tasks, such as interpreting medical records and providing accurate treatment recommendations [30,32,33]. The efficiency gains from LLMs in clinical settings must be balanced against their limitations and potential risks [108]. As these models continue to evolve, their role in mental health support is likely to expand, with a focus on enhancing data privacy, biases, and ethical considerations in clinical implementation. LLMs are trained on vast amounts of data, which may include sensitive personal and health information. Ensuring compliance with privacy regulations such as the general data protection regulation is essential to protect patient confidentiality [116,117]. The inadvertent inclusion of personally identifiable information in pretraining datasets can compromise patient privacy, and LLMs can make privacy-invading inferences from seemingly innocuous data. Implementing measures such as data anonymization and safe data storage procedures is crucial to address these issues. Biases in LLMs are another critical issue that must be considered. These models would perpetuate and magnify biases in their training data, leading to differential treatment and outcomes, particularly in populations considered vulnerable [118]. For instance, biases in gender, race, and socioeconomic status can result in inaccurate or misleading information, which may exacerbate existing health disparities [119]. To mitigate these risks, it is important to develop techniques for identifying, alleviating, and preventing biases in LLMs. Ethical concerns are paramount when using LLMs in mental health. The potential for LLMs to generate false or misleading information is a particular concern in health care. Ensuring that LLMs are rigorously tested and monitored is crucial to maintaining reliability in patient care. In addition, transparency is vital for health care professionals to trust the results of LLMs. These LLMs should provide clear, understandable insights for operation with sufficient explanation.

### Prospective Future Directions of LLMs in Mental Health

In the future, the application trend of LLMs in mental health is expected to continue to rise, and the aspects of their applications will be broader. Initially, most studies based on the LLMs with textual data and multimodal data, such as pictures, videos, and sounds, could be integrated with multimodal LLMs. Various data types might further improve the performance of mental disorders’ detection and identification. Several studies have explored multimodal LLMs in mental health research [72,108]. Moreover, existing studies mainly focus on depression and suicidality; more mental disorders should be investigated with LLMs, especially for rare mental disorders, such as borderline personality disorder and bipolar disorder. The applications of LLMs in treatments, interventions, and preliminary care would be beneficial for these patients. Furthermore, although several studies have developed chatbots for early detection or intervention in mental disorders, further research is needed to enhance the accuracy and robustness of LLMs. In addition, it is important to provide open-resource and fine-tuned LLMs for mental health, especially for low- and middle-income countries. Although LLMs show great performance in various applications in mental health, several areas of LLMs should be improved. First, there is a need for more high-quality, diverse, and representative datasets to train LLMs for mental health applications, ensuring that the models can understand and respond to a wide range of mental health–related queries and scenarios. Second, while LLMs can generate coherent and contextually appropriate responses, they still lag behind human performance in terms of empathy and emotional intelligence, which are crucial in mental health support. Third, LLMs need to improve their ability to reason and understand the context and nuances of mental health dialogues, which often require a deep understanding of human emotions and psychological states.

Finally, it is important to establish standards for privacy, safety, and ethical considerations when LLMs process sensitive personal and health information [120,121]. These standards are essential to mitigate the potential risks and ensure the responsible use of LLMs in health care settings. On the one hand, the model training process for LLMs inevitably involves the collection of stigmatizing or biased data [118], which can generate hallucinatory content that may mislead or harm patients. For instance, biased data can result in inaccurate or inappropriate medical advice, which could have serious consequences for individuals seeking health information [105]. Several studies underscore the risk of sensitive data exposure and emphasize the prevention of harmful content generation [71,73]. On the other hand, the convenience and low cost of LLMs may lead teenagers to become overly dependent on them for mental support. This excessive reliance could result in several negative outcomes. It may lead to addiction to the internet-based world, negatively impacting their daily lives and social interactions. Moreover, it could delay the optimal time for teenagers to seek professional help, potentially exacerbating their mental health issues. While LLMs can provide initial support and guidance, they cannot replace the nuanced understanding and empathy that human professionals can offer. Furthermore, LLMs cannot address crises effectively [122]. Although they can identify extreme emotions during conversations, they typically only suggest seeking professional assistance without providing direct and effective measures. LLMs lack the clinical judgment required to handle emergencies, which means they cannot offer the immediate support that may be necessary in critical situations. Therefore, in the practical application of LLMs, professional intervention is essential [123]. Experts should develop a dedicated system of ethical standards to guide the use of LLMs in health care. This system should include regular supervision and evaluations to ensure that LLMs are used responsibly and ethically. LLMs should be used as supplementary tools rather than complete replacements for human roles. They can provide initial support and guidance, but ultimately, the responsibility for clinical judgment and patient care should remain with trained health care professionals. Future advancements depend on collaborative efforts to refine technology, develop standardized evaluations, and ensure ethical applications, aiming to realize LLMs’ full potential in supporting mental health care.

### Comparison With Previous Studies

Several reviews have explored the applications of LLMs in mental health care from various perspectives. A viewpoint article and a preprint summarized the opportunities and risks associated with using LLMs in mental health [115,124]. The viewpoint article focuses on application scenarios, including education, assessment, and intervention, while the preprint clarifies the potential opportunities and risks of LLMs from an ecological conceptualization perspective, encompassing the care seeker, caregiver, institution, and society [124]. The analysis methods and application categories in these reviews differ from those in this scoping review. Another preprint scoping review also identified diverse applications of LLMs in mental health care, including preprint articles [25]. On the basis of 34 articles screened from 313 articles published between October 1, 2019, and December 2, 2023, the researchers categorized the application areas into 3 domains: the deployment of conversational agents, resource enrichment, and detailed diagnosis classification. However, this scoping review delved deeper into LLMs and training techniques, dataset characteristics, validation measures and metrics, and challenges. In contrast, our study offers a more targeted and comprehensive reference guide for LLM applications in mental health. Specifically, we compare the application effects of LLMs with human evaluations and among different models, aiming to provide a detailed and structured overview of LLMs’ performance and potential in mental health scenarios. Another systematic review with 40 articles assessed the clinical applications of LLMs in mental health [26], focusing on their potential and inherent risks. Although this review summarized the results of each article in tabular form, it did not extract key information. Our scoping review searched 4 English language databases and 3 Chinese language databases with more specific search terms and a wider time range. We categorized these applications into 3 key areas: screening or detection of mental disorders, supporting clinical treatments and interventions, and assisting in mental health counseling and education based on 95 articles screened from 4859 articles. We also extracted key information about LLMs’ applications and developed frameworks for each application area. These frameworks are designed to help researchers and clinicians understand the use of LLMs in mental health, even without a background in artificial intelligence. Furthermore, we provided the fine-tuned LLMs for mental health and compared the advantages and disadvantages of LLMs. This comparative approach offers a more nuanced understanding of how different models and human expertise can be leveraged in mental health applications, aiding researchers and clinicians in selecting suitable LLMs for their specific mental health tasks.

Although this scoping review explored the applications of LLMs in mental health, several limitations should be considered. First, there is an absence of assessment of the risk of bias due to the unique nature of these included studies. Moreover, we cannot perform a meta-analysis due to the diversity of methods and tasks in the included studies. Second, with the rapid development of LLMs, the results of comparative studies would be cautious. The performance of these LLMs may have significantly improved. Third, the preprint studies (eg, from arXiv and medRxiv platforms) were not included in this review due to the lack of peer review, though they were published recently. Finally, this review is limited to studies published in English and Chinese, which may compromise the comprehensiveness and representativeness of the findings. This approach may overlook the applications and contributions of LLMs in other regions that speak different languages, thereby failing to provide a comprehensive global overview of LLM applications in mental health. In addition, different languages present unique challenges and opportunities for LLM applications in mental health. Although this scoping review identified a limited number of studies applying LLMs for depression detection in Malay dialects [41] and developing therapeutic dialogue agents in the Polish language [106], the performance of LLMs across various languages can vary based on linguistic characteristics, data availability, and cultural contexts. Limiting the review to English and Chinese studies may underestimate these differences. Moreover, low- and middle-income regions, which often rely on technologies such as LLMs to enhance mental health service accessibility and quality, may use languages other than English or Chinese. Limiting the review to English and Chinese studies could neglect the actual needs and potential contributions of these regions, thereby affecting the development and application of LLMs in those areas. To provide a more holistic view of LLM applications in mental health, future scoping reviews should consider a broader range of languages to provide a more comprehensive understanding.

### Conclusions

This scoping review summarized the applications of LLMs in the mental health field, including trends, application areas, performance comparisons, challenges, and future directions. The applications mainly focused on 3 areas: screening or detection of mental disorders, supporting clinical treatments and interventions, and assisting in mental health counseling and education. Compared with nontransformer models and human experts, LLMs demonstrate higher capabilities in information acquisition and analysis, generating natural language responses, and addressing complex reasoning problems. As these models continue to evolve, their role in mental health support is likely to expand, with a focus on enhancing accuracy, sensitivity, and ethical considerations in clinical implementation.

## Data Availability

All data generated or analyzed during this study are included in this published article and its supplementary information files. For detailed data, readers may refer to the original studies cited in our review. The complete list of the included studies can be found in the References section.

## Appendix

Multimedia Appendix 1 PRISMA-ScR checklist.

Multimedia Appendix 2 Search terms used in the main review for English-language databases.

Multimedia Appendix 3 Search terms used in the main review for Chinese-language databases.

Multimedia Appendix 4 List of studies excluded at the full-text screening stage.

Multimedia Appendix 5 Higher resolution versions of Figures 2-4.

Multimedia Appendix 6 Categorization of articles based on mental health applications across 95 studies.

Multimedia Appendix 7 The metrics results of performance between large language models and humans.

Multimedia Appendix 8 Performance comparisons between various large language models.

## Abbreviations

EMR: electronic medical record
LLM: large language model
NLP: natural language processing
PaLM: Pathways Language Model
PRISMA-ScR: Preferred Reporting Items for Systematic Reviews and Meta-Analyses Extension for Scoping Reviews

## References

[1] Carswell K, Cuijpers P, Gray B, Kestel D, Malik A, Weissbecker I, van Ommeren M (2024). WHO recommendations on psychological interventions for mental disorders. Lancet Psychiatry.

[2] Sacco R, Camilleri N, Eberhardt J, Umla-Runge K, Newbury-Birch D (2024). A systematic review and meta-analysis on the prevalence of mental disorders among children and adolescents in Europe. Eur Child Adolesc Psychiatry.

[3] Brohan E, Chowdhary N, Dua T, Barbui C, Thornicroft G, Kestel D (2024). The WHO Mental Health Gap Action Programme for mental, neurological, and substance use conditions: the new and updated guideline recommendations. Lancet Psychiatry.

[4] Flett GL, Hewitt PL (2024). The need to focus on perfectionism in suicide assessment, treatment and prevention. World Psychiatry.

[5] Chang Y, Wang X, Wang J, Wu Y, Yang L, Zhu K, Chen H, Yi X, Wang C, Wang Y, Ye W, Zhang Y, Chang Y, Yu PS, Yang Q, Xie X (2024). A Survey on Evaluation of Large Language Models. ACM Trans Intell Syst Technol.

[6] Brown TB, Mann B, Ryder N, Subbiah M, Kaplan J, Dhariwal P, Neelakantan A, Shyam P, Sastry G, Askell A, Agarwal S, Herbert-Voss A, Krueger G, Henighan T, Child R, Ramesh A, Ziegler DM, Wu J, Winter C, Hesse C, Chen M, Sigler E, Litwin M, Gray S, Chess B, Clark J, Berner C, McCandlish S, Radford A, Sutskever I, Amodei D Language models are few-shot learners. arXiv.

[7] Chowdhery A, Narang S, Devlin J, Bosma M, Mishra G, Roberts A, Barham P, Chung HW, Sutton C, Gehrmann S, Schuh P, Shi K, Tsvyashchenko S, Maynez J, Rao A, Barnes P, Tay Y, Shazeer N, Prabhakaran V, Reif E, Du N, Hutchinson B, Pope R, Bradbury J, Austin J, Isard M, Gur-Ari G, Yin P, Duke T, Levskaya A, Ghemawat S, Dev S, Michalewski H, Garcia X, Misra V, Robinson K, Fedus L, Zhou D, Ippolito D, Luan D, Lim H, Zoph B, Spiridonov A, Sepassi R, Dohan D, Agrawal S, Omernick M, Dai AM, Pillai TS, Pellat M, Lewkowycz A, Moreira E, Child R, Polozov O, Lee K, Zhou Z, Wang X, Saeta B, Diaz M, Firat O, Catasta M, Wei J, Meier-Hellstern K, Eck D, Dean J, Petrov S, Fiedel N (2023). PaLM: scaling language modeling with pathways. J Mach Learn Res.

[8] Touvron H, Lavril T, Izacard G, Martinet X, Lachaux MA, Lacroix T, Rozière B, Goyal N, Hambro E, Azhar F, Rodriguez A, Joulin A, Grave E, Lample G LLaMA: open and efficient foundation language models. arXiv.

[9] Zhao WX, Zhou K, Li J, Tang T, Wang X, Hou Y, Min Y, Zhang B, Zhang J, Dong Z, Du Y, Yang C, Chen Y, Chen Z, Jiang J, Ren R, Li Y, Tang X, Liu Z, Liu P, Nie JY, Wen JRC A survey of large language models. arXiv.

[10] Clusmann J, Kolbinger FR, Muti HS, Carrero ZI, Eckardt JN, Laleh NG, Löffler CM, Schwarzkopf SC, Unger M, Veldhuizen GP, Wagner SJ, Kather JN (2023). The future landscape of large language models in medicine. Commun Med (Lond).

[11] Gao J, Lin CY (2004). Introduction to the special issue on statistical language modeling. ACM Trans Asian Lang Inf Process.

[12] Kombrink S, Mikolov T, Karafiát M, Burget L (2011). Recurrent neural network based language modeling in meeting recognition. Proceedings of the Interspeech 2011.

[13] Torous J, Blease C (2024). Generative artificial intelligence in mental health care: potential benefits and current challenges. World Psychiatry.

[14] Beredo JL, Ong EC (2022). A hybrid response generation model for an empathetic conversational agent. Proceedings of the 2022 International Conference on Asian Language Processing.

[15] Wu Y, Chen J, Mao K, Zhang Y (2023). Automatic post-traumatic stress disorder diagnosis via clinical transcripts: a novel text augmentation with large language models. Proceedings of the 2023 IEEE Biomedical Circuits and Systems Conference.

[16] Bartal A, Jagodnik KM, Chan SJ, Dekel S (2024). AI and narrative embeddings detect PTSD following childbirth via birth stories. Sci Rep.

[17] Lee C, Mohebbi M, O'Callaghan E, Winsberg M (2024). Large language models versus expert clinicians in crisis prediction among telemental health patients: comparative study. JMIR Ment Health.

[18] Jeon E, Yoon N, Sohn SY (2023). Exploring new digital therapeutics technologies for psychiatric disorders using BERTopic and PatentSBERTa. Technol Forecast Soc Change.

[19] Mármol-Romero AM, García-Vega M, García-Cumbreras MÁ, Montejo-Ráez A (2024). An empathic GPT-based chatbot to talk about mental disorders with Spanish teenagers. Int J Hum Comput Interact.

[20] Elyoseph Z, Levkovich I, Shinan-Altman S (2024). Assessing prognosis in depression: comparing perspectives of AI models, mental health professionals and the general public. Fam Med Community Health.

[21] Perlis RH, Goldberg JF, Ostacher MJ, Schneck CD (2024). Clinical decision support for bipolar depression using large language models. Neuropsychopharmacology.

[22] He W, Zhang W, Jin Y, Zhou Q, Zhang H, Xia Q (2024). Physician versus large language model chatbot responses to web-based questions from autistic patients in Chinese: cross-sectional comparative analysis. J Med Internet Res.

[23] Metzler H, Baginski H, Niederkrotenthaler T, Garcia D (2022). Detecting potentially harmful and protective suicide-related content on Twitter: machine learning approach. J Med Internet Res.

[24] Xu X, Yao B, Dong Y, Gabriel S, Yu H, Hendler J, Ghassemi M, Dey AK, Wang D (2024). Mental-LLM: leveraging large language models for mental health prediction via online text data. Proc ACM Interact Mob Wearable Ubiquitous Technol.

[25] Hua Y, Liu F, Yang K, Li Z, Na H, Sheu YH, Zhou P, Moran LV, Ananiadou S, Beam A, Torous J Large language models in mental health care: a scoping review. arXiv.

[26] Guo Z, Lai A, Thygesen JH, Farrington J, Keen T, Li K (2024). Large language models for mental health applications: systematic review. JMIR Ment Health.

[27] (2024). The applications of large language models in mental health: a scoping review. OSF Registries.

[28] Armstrong R, Hall BJ, Doyle J, Waters E (2011). Cochrane update. 'Scoping the scope' of a Cochrane review. J Public Health (Oxf).

[29] Munn Z, Peters MD, Stern C, Tufanaru C, McArthur A, Aromataris E (2018). Systematic review or scoping review? Guidance for authors when choosing between a systematic or scoping review approach. BMC Med Res Methodol.

[30] Yang K, Zhang T, Kuang Z, Xie Q, Huang J, Ananiadou S (2024). MentaLLaMA: interpretable mental health analysis on social media with large language models. Proceedings of the ACM Web Conference 2024.

[31] Vajre V, Naylor M, Kamath U, Shehu A (2021). PsychBERT: a mental health language model for social media mental health behavioral analysis. Proceedings of the 2021 IEEE International Conference on Bioinformatics and Biomedicine.

[32] Zhang Y, Lyu H, Liu Y, Zhang X, Wang Y, Luo J (2021). Monitoring depression trends on Twitter during the COVID-19 pandemic: observational study. JMIR Infodemiology.

[33] Suri M, Semwal N, Chaudhary D, Gorton I, Kumar B (2022). I don’t feel so good! Detecting depressive tendencies using transformer-based multimodal frameworks. Proceedings of the 2022 5th International Conference on Machine Learning and Natural Language Processing.

[34] Guo Y, Liu J, Wang L, Qin W, Hao S, Hong R (2024). A prompt-based topic-modeling method for depression detection on low-resource data. IEEE Trans Comput Soc Syst.

[35] Abilkaiyrkyzy A, Laamarti F, Hamdi M, Saddik AE (2024). Dialogue system for early mental illness detection: toward a digital twin solution. IEEE Access.

[36] Abdullah M, Negied N (2024). Detection and prediction of future mental disorder from social media data using machine learning, ensemble learning, and large language models. IEEE Access.

[37] Tao Y, Yang M, Shen H, Yang Z, Weng Z, Hu B (2023). Classifying anxiety and depression through LLMs virtual interactions: a case study with ChatGPT. Proceedings of the 2023 IEEE International Conference on Bioinformatics and Biomedicine.

[38] Sood P, Yang X, Wang P (2023). Enhancing depression detection from narrative interviews using language models. Proceedings of the IEEE International Conference on Bioinformatics and Biomedicine.

[39] Lu KC, Thamrin SA, Chen AL (2023). Depression detection via conversation turn classification. Multimed Tools Appl.

[40] Bokolo BG, Liu Q (2023). Deep learning-based depression detection from social media: comparative evaluation of ML and transformer techniques. Electronics.

[41] Hayati MF, Ali MA, Rosli AN (2022). Depression detection on Malay dialects using GPT-3. Proceedings of the 2022 IEEE-EMBS Conference on Biomedical Engineering and Sciences.

[42] Danner M, Hadzic B, Gerhardt S, Ludwig S, Uslu I, Shao P (2023). Advancing mental health diagnostics: GPT-based method for depression detection. Proceedings of the 62nd Annual Conference of the Society of Instrument and Control Engineers.

[43] Wang X, Chen S, Li T, Li W, Zhou Y, Zheng J, Chen Q, Yan J, Tang B (2020). Depression risk prediction for Chinese microblogs via deep-learning methods: content analysis. JMIR Med Inform.

[44] Owen D, Antypas D, Hassoulas A, Pardiñas AF, Espinosa-Anke L, Collados JC (2023). Enabling early health care intervention by detecting depression in users of web-based forums using language models: longitudinal analysis and evaluation. JMIR AI.

[45] Senn S, Tlachac ML, Flores R, Rundensteiner E (2022). Ensembles of BERT for depression classification. Proceedings of the 44th Annual International Conference of the IEEE Engineering in Medicine & Biology Society.

[46] Sezgin E, Chekeni F, Lee J, Keim S (2023). Clinical accuracy of large language models and Google search responses to postpartum depression questions: cross-sectional study. J Med Internet Res.

[47] Sadeghi M, Egger B, Agahi R, Richer R, Capito K, Rupp LH (2023). Exploring the capabilities of a language model-only approach for depression detection in text data. Proceedings of the IEEE EMBS International Conference on Biomedical and Health Informatics.

[48] Farruque N, Goebel R, Sivapalan S, Zaïane OR (2024). Depression symptoms modelling from social media text: an LLM driven semi-supervised learning approach. Lang Resour Eval.

[49] Tey W, Goh H, Lim AH, Phang C (2023). Pre- and post-depressive detection using deep learning and textual-based features. Int J Technol.

[50] de Hond A, van Buchem M, Fanconi C, Roy M, Blayney D, Kant I, Steyerberg E, Hernandez-Boussard T (2024). Predicting depression risk in patients with cancer using multimodal data: algorithm development study. JMIR Med Inform.

[51] Ilias L, Mouzakitis S, Askounis D (2024). Calibration of transformer-based models for identifying stress and depression in social media. IEEE Trans Comput Soc Syst.

[52] Levkovich I, Elyoseph Z (2023). Identifying depression and its determinants upon initiating treatment: ChatGPT versus primary care physicians. Fam Med Community Health.

[53] Toto E, Tlachac ML, Rundensteiner EA (2021). AudiBERT: a deep transfer learning multimodal classification framework for depression screening. Proceedings of the 30th ACM International Conference on Information & Knowledge Management.

[54] Lau C, Zhu X, Chan WY (2023). Automatic depression severity assessment with deep learning using parameter-efficient tuning. Front Psychiatry.

[55] Verma S, Joshi RC, Dutta MK, Jezek S, Burget R, Vishal (2023). AI-enhanced mental health diagnosis: leveraging transformers for early detection of depression tendency in textual data. Proceedings of the 15th International Congress on Ultra Modern Telecommunications and Control Systems and Workshops.

[56] Pourkeyvan A, Safa R, Sorourkhah A (2024). Harnessing the power of hugging face transformers for predicting mental health disorders in social networks. IEEE Access.

[57] Englhardt Z, Ma C, Morris ME, Chang CC, Xu X, Qin L, McDuff D, Liu X, Patel S, Iyer V (2024). From classification to clinical insights: towards analyzing and reasoning about mobile and behavioral health data with large language models. Proc ACM Interact Mob Wearable Ubiquitous Technol.

[58] Firoz N, Berestneva O, Aksyonov SV (2024). Dual layer Cogni - insight deep-mood encoder: a two- tiered approach for depression detection. Proceedings of the International Russian Smart Industry Conference.

[59] Huang L (2024). A study on the design of a depression diagnostic framework based on DepGPT and neural networks. Proceedings of the 2nd International Conference on Mechatronics, IoT and Industrial Informatics.

[60] Shin D, Kim H, Lee S, Cho Y, Jung W (2024). Using large language models to detect depression from user-generated diary text data as a novel approach in digital mental health screening: instrument validation study. J Med Internet Res.

[61] Qorich M, El Ouazzani R (2024). Advanced deep learning and large language models for suicide ideation detection on social media. Prog Artif Intell.

[62] Howard D, Maslej MM, Lee J, Ritchie J, Woollard G, French L (2020). Transfer learning for risk classification of social media posts: model evaluation study. J Med Internet Res.

[63] Levkovich I, Elyoseph Z (2023). Suicide risk assessments through the eyes of ChatGPT-3.5 versus ChatGPT-4: vignette study. JMIR Ment Health.

[64] Ghanadian H, Nejadgholi I, Osman HA (2024). Socially aware synthetic data generation for suicidal ideation detection using large language models. IEEE Access.

[65] Diniz EJ, Fontenele JE, de Oliveira AC, Bastos VH, Teixeira S, Rabêlo RL, Calçada DB, Dos Santos RM, de Oliveira AK, Teles AS (2022). Boamente: a natural language processing-based digital phenotyping tool for smart monitoring of suicidal ideation. Healthcare (Basel).

[66] Wang R, Yang BX, Ma Y, Wang P, Yu Q, Zong X, Huang Z, Ma S, Hu L, Hwang K, Liu Z (2021). Medical-level suicide risk analysis: a novel standard and evaluation model. IEEE Internet Things J.

[67] Bauer B, Norel R, Leow A, Rached ZA, Wen B, Cecchi G (2024). Using large language models to understand suicidality in a social media-based taxonomy of mental health disorders: linguistic analysis of Reddit posts. JMIR Ment Health.

[68] Kumar A, Trueman TE, Cambria E (2022). Stress identification in online social networks. Proceedings of the IEEE International Conference on Data Mining Workshops.

[69] Jain B, Goyal G, Sharma M (2024). Evaluating emotional detection and classification capabilities of GPT-2 and GPT-neo using textual data. Proceedings of the 14th International Conference on Cloud Computing, Data Science & Engineering.

[70] Teferra BG, Rose J (2023). Predicting generalized anxiety disorder from impromptu speech transcripts using context-aware transformer-based neural networks: model evaluation study. JMIR Ment Health.

[71] Kim J, Leonte KG, Chen ML, Torous JB, Linos E, Pinto A, Rodriguez CI (2024). Large language models outperform mental and medical health care professionals in identifying obsessive-compulsive disorder. NPJ Digit Med.

[72] Gargari OK, Fatehi F, Mohammadi I, Firouzabadi SR, Shafiee A, Habibi G (2024). Diagnostic accuracy of large language models in psychiatry. Asian J Psychiatr.

[73] Wang Y, Yu Y, Liu Y, Ma Y, Pang PC (2023). Predicting patients' satisfaction with mental health drug treatment using their reviews: unified interchangeable model fusion approach. JMIR Ment Health.

[74] Hadar-Shoval D, Elyoseph Z, Lvovsky M (2023). The plasticity of ChatGPT's mentalizing abilities: personalization for personality structures. Front Psychiatry.

[75] Tanana MJ, Soma CS, Kuo PB, Bertagnolli NM, Dembe A, Pace BT, Srikumar V, Atkins DC, Imel ZE (2021). How do you feel? Using natural language processing to automatically rate emotion in psychotherapy. Behav Res Methods.

[76] Wan C, Ge X, Wang J, Zhang X, Yu Y, Hu J, Liu Y, Ma H (2022). Identification and impact analysis of family history of psychiatric disorder in mood disorder patients with pretrained language model. Front Psychiatry.

[77] Dai HJ, Su CH, Lee YQ, Zhang YC, Wang CK, Kuo CJ, Wu CS (2020). Deep learning-based natural language processing for screening psychiatric patients. Front Psychiatry.

[78] Gargari OK, Habibi G, Nilchian N, Shafiee A (2024). Comparative analysis of large language models in psychiatry and mental health: a focus on GPT, AYA, and Nemotron-3-8B. Asian J Psychiatr.

[79] Rathje S, Mirea DM, Sucholutsky I, Marjieh R, Robertson CE, Van Bavel JJ (2024). GPT is an effective tool for multilingual psychological text analysis. Proc Natl Acad Sci U S A.

[80] Lossio-Ventura JA, Weger R, Lee AY, Guinee EP, Chung J, Atlas L, Linos E, Pereira F (2024). A comparison of ChatGPT and fine-tuned open pre-trained transformers (OPT) against widely used sentiment analysis tools: sentiment analysis of COVID-19 survey data. JMIR Ment Health.

[81] Stigall W, Khan MA, Attota D, Nweke F, Pei Y (2024). Large language models performance comparison of emotion and sentiment classification. Proceedings of the 2024 ACM Southeast Conference.

[82] Yongsatianchot N, Torshizi PG, Marsella S (2023). Investigating large language models’ perception of emotion using appraisal theory. Proceedings of the 11th International Conference on Affective Computing and Intelligent Interaction Workshops and Demos.

[83] Goyal T, Rajeshbai DH, Gopalkrishna N, M T, HR M (2024). Mobile machine learning models for emotion and sarcasm detection in text: a solution for alexithymic individuals. Proceedings of the 3rd International Conference for Innovation in Technology.

[84] Elyoseph Z, Refoua E, Asraf K, Lvovsky M, Shimoni Y, Hadar-Shoval D (2024). Capacity of generative AI to interpret human emotions from visual and textual data: pilot evaluation study. JMIR Ment Health.

[85] Fan Y, Nie J, Sun X, Jiang X (2024). Exploring foundation models in detecting concerning daily functioning in psychotherapeutic context based on images from smart home devices. Proceedings of the IEEE International Workshop on Foundation Models for Cyber-Physical Systems & Internet of Things.

[86] Yang X, Chen A, PourNejatian N, Shin HC, Smith KE, Parisien C, Compas C, Martin C, Costa AB, Flores MG, Zhang Y, Magoc T, Harle CA, Lipori G, Mitchell DA, Hogan WR, Shenkman EA, Bian J, Wu Y (2022). A large language model for electronic health records. NPJ Digit Med.

[87] Huang S, Fu F, Yang K, Zhang K, Yang F (2024). Empowerment of large language models in psychological counseling through prompt engineering. Proceedings of the 2024 IEEE 4th International Conference on Software Engineering and Artificial Intelligence.

[88] Adhikary PK, Srivastava A, Kumar S, Singh SM, Manuja P, Gopinath JK, Krishnan V, Gupta SK, Deb KS, Chakraborty T (2024). Exploring the efficacy of large language models in summarizing mental health counseling sessions: benchmark study. JMIR Ment Health.

[89] Hodson N, Williamson S (2024). Can large language models replace therapists? Evaluating performance at simple cognitive behavioral therapy tasks. JMIR AI.

[90] McElroy E, Wood T, Bond R, Mulvenna M, Shevlin M, Ploubidis GB, Hoffmann MS, Moltrecht B (2024). Using natural language processing to facilitate the harmonisation of mental health questionnaires: a validation study using real-world data. BMC Psychiatry.

[91] Franco D'Souza R, Amanullah S, Mathew M, Surapaneni KM (2023). Appraising the performance of ChatGPT in psychiatry using 100 clinical case vignettes. Asian J Psychiatr.

[92] Wang X, Liu K, Wang C (2023). Knowledge-enhanced pre-training large language model for depression diagnosis and treatment. Proceedings of the IEEE 9th International Conference on Cloud Computing and Intelligent Systems.

[93] Friedman SF, Ballentine G (2024). Trajectories of sentiment in 11,816 psychoactive narratives. Hum Psychopharmacol.

[94] Kamoji S, Rozario S, Almeida S, Patil S, Patankar S, Pendhari H (2024). Mental health prediction using machine learning models and large language model. Proceedings of the 2024 Second International Conference on Inventive Computing and Informatics.

[95] Heston T (2023). Safety of large language models in addressing depression. Cureus.

[96] Llanes-Jurado J, Gómez-Zaragozá L, Minissi ME, Alcañiz M, Marín-Morales J (2024). Developing conversational virtual Humans for social emotion elicitation based on large language models. Expert Syst Appl.

[97] Berrezueta-Guzman S, Kandil M, Martin-Ruiz ML, de la Cruz IP, Krusche S (2024). Exploring the efficacy of robotic assistants with ChatGPT and Claude in enhancing ADHD therapy: innovating treatment paradigms. Proceedings of the International Conference on Intelligent Environments.

[98] Gabor-Siatkowska K, Sowański M, Rzatkiewicz R, Stefaniak I, Kozłowski M, Janicki A (2023). AI to train AI: using ChatGPT to improve the accuracy of a therapeutic dialogue system. Electronics.

[99] Maples B, Cerit M, Vishwanath A, Pea R (2024). Loneliness and suicide mitigation for students using GPT3-enabled chatbots. Npj Ment Health Res.

[100] Wu Y, Mao K, Zhang Y, Chen J (2024). CALLM: enhancing clinical interview analysis through data augmentation with large language models. IEEE J Biomed Health Inform.

[101] Cai Z, Fang H, Liu J, Xu G, Long Y (2024). Instruction tuning of LLM for unified information extraction in mental health domain. J Chin Inf Process.

[102] Aygün İ, Kaya M (2024). Use of large language models for medical synthetic data generation in mental illness. IET Conf Proc.

[103] Sung CW, Lee YK, Tsai YT (2024). A new pipeline for generating instruction dataset via RAG and self fine-tuning. Proceedings of the 2024 IEEE 48th Annual Computers, Software, and Applications Conference.

[104] Crasto R, Dias L, Miranda D, Kayande D (2021). CareBot: a mental health ChatBot. Proceedings of the 2nd International Conference for Emerging Technology.

[105] Ma Z, Mei Y, Su Z (2023). Understanding the benefits and challenges of using large language model-based conversational agents for mental well-being support. AMIA Annu Symp Proc.

[106] Zygadlo A (2021). A therapeutic dialogue agent for Polish language. Proceedings of the 9th International Conference on Affective Computing and Intelligent Interaction Workshops and Demos.

[107] Kumar H, Wang Y, Shi J, Musabirov I, Farb NA, Williams JJ (2023). Exploring the use of large language models for improving the awareness of mindfulness. Proceedings o the Extended Abstracts of the 2023 CHI Conference on Human Factors in Computing Systems.

[108] Thapa S, Adhikari S (2024). GPT-4o and multimodal large language models as companions for mental wellbeing. Asian J Psychiatr.

[109] Grabb D (2023). The impact of prompt engineering in large language model performance: a psychiatric example. J Med Artif Intell.

[110] Milligan G, Bernard A, Dowthwaite L, Vallejos EP, Davis J, Salhi L, Goulding J (2024). Developing a single‐session outcome measure using natural language processing on digital mental health transcripts. Couns Psychother Res.

[111] Spallek S, Birrell L, Kershaw S, Devine EK, Thornton L (2023). Can we use ChatGPT for mental health and substance use education? Examining its quality and potential harms. JMIR Med Educ.

[112] Wang L, Chen X, Deng X, Wen H, You M, Liu W, Li Q, Li J (2024). Prompt engineering in consistency and reliability with the evidence-based guideline for LLMs. NPJ Digit Med.

[113] Lainwright N, Pemberton M Assessing the response strategies of large language models under uncertainty: a comparative study using prompt engineering. OSF Preprints.

[114] Hadi MU, Tashi QA, Qureshi R, Shah A, Irfan M, Zafar A, Shaikh M, Akhtar N, Hassan SZ, Shoman M, Wu J, Mirjalili S, Shah M A survey on large language models: applications, challenges, limitations, and practical usage. TechRxiv.

[115] Lawrence HR, Schneider RA, Rubin SB, Matarić MJ, McDuff DJ, Jones Bell M (2024). The opportunities and risks of large language models in mental health. JMIR Ment Health.

[116] Neame R (2014). Privacy protection for personal health information and shared care records. Inform Prim Care.

[117] Paavola J, Ekqvist J (2017). Privacy preserving and resilient cloudified IoT architecture to support eHealth systems. Proceedings of the Third International Conference on Interoperability, Safety and Security in IoT.

[118] Alber DA, Yang Z, Alyakin A, Yang E, Rai S, Valliani AA, Zhang J, Rosenbaum GR, Amend-Thomas AK, Kurland DB, Kremer CM, Eremiev A, Negash B, Wiggan DD, Nakatsuka MA, Sangwon KL, Neifert SN, Khan HA, Save AV, Palla A, Grin EA, Hedman M, Nasir-Moin M, Liu XC, Jiang LY, Mankowski MA, Segev DL, Aphinyanaphongs Y, Riina HA, Golfinos JG, Orringer DA, Kondziolka D, Oermann EK (2025). Medical large language models are vulnerable to data-poisoning attacks. Nat Med.

[119] Potter L, Zawadzki MJ, Eccleston CP, Cook JE, Snipes SA, Sliwinski MJ, Smyth JM (2019). The intersections of race, gender, age, and socioeconomic status: implications for reporting discrimination and attributions to discrimination. Stigma Health.

[120] Haupt CE, Marks M (2023). AI-generated medical advice-GPT and beyond. JAMA.

[121] Freyer O, Wiest IC, Kather JN, Gilbert S (2024). A future role for health applications of large language models depends on regulators enforcing safety standards. Lancet Digit Health.

[122] Han T, Kumar A, Agarwal C, Lakkaraju H Towards safe large language models for medicine. ArXiv.

[123] Fu G, Zhao Q, Dan L, Li J, Song J, Wei Z, Liu S, Wang F, Wang Y, Cheng L, Zhang J, Yang BY Enhancing psychological counseling with large language model: a multifaceted decision-support system for non-professionals. arXiv.

[124] De Choudhury M, Pendse SR, Kumar N Benefits and harms of large language models in digital mental health. PsyArXiv Preprints.

