# Supplementary material for: The Applications of Large Language Models in Mental Health: Scoping Review
Source: J Med Internet Res. 2025 May 5;27:e69284. doi: 10.2196/69284 (PMC12089884; doi:10.2196/69284)
Supplement: Multimedia Appendix 2 [file jmir_v27i1e69284_app2.docx]

Supplemental Files

Table S2. Search terms used in the main review for English-language databases.

|  | Pubmed | Web of science | IEEE Xplore | Cochrane library |
| --- | --- | --- | --- | --- |
| 1. Search terms |  |  |  |  |
|  | (((psychiatr*[Title/Abstract])OR(mental health)OR(depress*)OR(anxiety)OR(posttraumatic stress disorder)OR(PTSD)OR(bipolar disorder)OR(schizophrenia)OR(obsessive-compulsive disorder)OR(personality disorder)OR(insomnia)OR(suicid*)) AND ((large language model[Title/Abstract])OR(OpenAI language model)OR(generative AI)OR(generative artificial intelligence)OR(BERT)OR(GPT) AND (2019/01/01:2024/08/31[dp]))) | (TS=((((psychiatr*)OR(mental health)OR(depress*)OR(anxiety)OR(posttraumatic stress disorder)OR(PTSD)OR(bipolar disorder)OR(schizophrenia)OR(obsessive-compulsive disorder)OR(personality disorder)OR(insomnia)OR(suicid*)))) AND TS=((large language model)OR(OpenAI language model)OR(generative AI)OR(generative artificial intelligence)OR(BERT)OR(GPT))) NOT (SILOID==("PPRN")) | ("mental health" OR psychiatri* OR depress* OR anxiety OR "posttraumatic stress disorder" OR PTSD OR "bipolar disorder" OR schizophrenia OR "obsessive-compulsive disorder" OR "personality disorder" OR insomnia OR suicid*) AND ("large language model" OR "OpenAI language model" OR "generative AI" OR BERT OR GPT) | (Title Abstract Keyword  = (((psychiatr*) OR(mental health)OR(depress*)OR(anxiety)OR(posttraumatic stress disorder)OR(PTSD)OR(bipolar disorder)OR(schizophrenia)OR(obsessive-compulsive disorder)OR(personality disorder)OR(insomnia)OR(suicid*)) AND ((large language model) OR(OpenAI language model)OR(generative AI)OR(generative artificial intelligence)OR(BERT)OR(GPT) |
| 2. Range |  |  |  |  |
|  | 2019/01/01 to 2024/08/31 | 2019/01/01 to 2024/08/31 | 2019/01/01 to 2024/08/31 | 2019/01 to 2024/08 |
| 3. Final Search Results |  |  |  |  |
|  | 2,326 | 1,806 | 193 | 202 |
