# Supplementary material for: The Applications of Large Language Models in Mental Health: Scoping Review
Source: J Med Internet Res. 2025 May 5;27:e69284. doi: 10.2196/69284 (PMC12089884; doi:10.2196/69284)
Supplement: Multimedia Appendix 3 [file jmir_v27i1e69284_app3.docx]

Supplemental Files

Table S3. Search terms used in the main review for Chinese-language databases.

|  | CNKI | Weipu | Wanfang |
| --- | --- | --- | --- |
| 1. Search terms |  |  |  |
|  | ("大语言模型" OR "生成式人工智能" OR "GPT" OR "自然语言处理")  AND ("心理健康评估" OR "心理障碍诊断" OR "精神疾病筛查")  AND ("情感分析" OR "心理状态预测") | ("大语言模型" OR "生成式人工智能" OR "GPT" OR "自然语言处理")  AND ("心理健康评估" OR "心理障碍诊断" OR "精神疾病筛查")  AND ("情感分析" OR "心理状态预测") | ("大语言模型" OR "生成式人工智能" OR "GPT" OR "自然语言处理")  AND ("心理健康评估" OR "心理障碍诊断" OR "精神疾病筛查")  AND ("情感分析" OR "心理状态预测") |
| 2. Range |  |  |  |
|  | 2019/01/01 to 2024/08/31 | 2019 to 2024 | 2019 to 2024 |
| 3. Final Search Results |  |  |  |
|  | 88 | 26 | 218 |
