# Supplementary material for: The Applications of Large Language Models in Mental Health: Scoping Review
Source: J Med Internet Res. 2025 May 5;27:e69284. doi: 10.2196/69284 (PMC12089884; doi:10.2196/69284)
Supplement: Multimedia Appendix 4 [file jmir_v27i1e69284_app4.docx]

Supplemental Files

Table S4. List of studies excluded at the full-text screening stage.

|  | Title | Reference | Exclusion reason |
| --- | --- | --- | --- |
| 1 | How do you feel? Using natural language processing to automatically rate emotion in psychotherapy | (Tanana et al., 2021) | It’s a preprint paper. |
| 2 | Bias Against 93 Stigmatized Groups in Masked Language Models and Downstream Sentiment Classification Tasks | (Mei et al., 2023) | It’s a preprint paper. |
| 3 | LAXARY: A Trustworthy Explainable Twitter Analysis Model for Post-Traumatic Stress Disorder Assessment | (Ul Alam et al., 2020) | It’s a preprint paper. |
| 4 | Few-shot Language Coordination by Modeling Theory of Mind | (Zhu et al., 2021) | It’s a preprint paper. |
| 5 | Exploring the Efficacy of Robotic Assistants with ChatGPT and Claude in Enhancing ADHD Therapy: Innovating Treatment Paradigms | (Berrezueta-Guzman et al., 2024) | It’s a preprint paper. |
| 6 | Large Language Models Improve Alzheimer's Disease Diagnosis Using Multi-Modality Data | (Feng et al., 2023) | It’s a preprint paper. |
| 7 | Chain-of-Interaction: Enhancing Large Language Models for Psychiatric Behavior Understanding by Dyadic Contexts | (Han et al., 2024) | It’s a preprint paper. |
| 8 | SAPIEN: Affective Virtual Agents Powered by Large Language Models* | (Hasan et al., 2023) | It’s a preprint paper. |
| 9 | Empirical Evaluation of Pre-trained Transformers for Human-Level NLP: The Role of Sample Size and Dimensionality | (Ganesan et al., 2021) | It’s a preprint paper. |
| 10 | GLUCOSE: GeneraLized and COntextualized Story Explanations | (Mostafazadeh et al., 2020) | It’s a preprint paper. |
| 11 | The usefulness of ChatGPT for psychotherapists and patients | (Raile, 2024) | It’s a preprint paper. |
| 12 | Cognitive Reframing of Negative Thoughts through Human-Language Model Interaction | (Sharma et al., 2023) | It’s a preprint paper. |
| 13 | PsyQA: A Chinese Dataset for Generating Long Counseling Text for Mental Health Support | (Sun et al., 2021) | It’s a preprint paper. |
| 14 | Using ChatGPT to promote college students' participation in physical activities and its effect on mental health | (Zhang and Liu, 2024) | It’s a preprint paper. |
| 15 | The Artificial Third: A Broad View of the Effects of Introducing Generative Artificial Intelligence on Psychotherapy | (Haber et al., 2024) | It’s a review. |
| 16 | ChatGPT on ECT: Can Large Language Models Support Psychoeducation? | (Lundin et al., 2023) | It’s a review. |
| 17 | Large language models could change the future of behavioral healthcare: a proposal for responsible development and evaluation | (Stade et al., 2024) | It’s a review. |
| 18 | ChatGPT and mental healthcare: balancing benefits with risks of harms | (Blease & Torous, 2023) | It’s a review. |
| 19 | Comparing the Perspectives of Generative AI, Mental Health Experts, and the General Public on Schizophrenia Recovery: Case Vignette Study | (Elyoseph & Levkovich, 2024) | It’s a review. |
| 20 | The Role of Humanization and Robustness of Large Language Models in Conversational Artificial Intelligence for Individuals With Depression: A Critical Analysis | (Ferrario et al., 2024) | It’s a review. |
| 21 | Large Language Models and Healthcare Alliance: Potential and Challenges of Two Representative Use Cases | (García-Méndez & de Arriba-Pérez, 2024) | It’s a review. |
| 22 | Can Large Language Models Replace Therapists? Evaluating Performance at Simple Cognitive Behavioral Therapy Tasks | (Hodson & Williamson, 2024) | It’s a review. |
| 23 | Beyond rating scales: With targeted evaluation, large language models are poised for psychological assessment | (Kjell et al., 2024) | It’s a review. |
| 24 | The Opportunities and Risks of Large Language Models in Mental Health | (Lawrence et al., 2024) | It’s a review. |
| 25 | Clinical Accuracy of Large Language Models and Google Search Responses to Postpartum Depression Questions: Cross-Sectional Study | (Sezgin et al., 2023) | It’s a review. |
| 26 | Large language models in psychiatry: Opportunities and challenges | (Volkmer et al., 2024) | It’s a review. |
| 27 | ChatGPT: Opportunities, risks and priorities for psychiatry | (Wei et al., 2023) | It’s a review. |
| 28 | Psychological insights into the research and practice of embodied conversational agents, chatbots and social assistive robots: a systematic meta-review | (Kiuchi et al., 2023) | It’s a review. |
| 29 | Prompt engineering for digital mental health: a short review | (Priyadarshana et al., 2024) | It’s a review. |
| 30 | Development of multimodal sentiment recognition and understanding | (Tao et al., 2024) | It’s a review. |
| 31 | An Integrative Survey on Mental Health Conversational Agents to Bridge Computer Science and Medical Perspectives | (Cho et al., 2023) | It's a review. |
| 32 | An Introduction to Generative Artificial Intelligence in Mental Health Care: Considerations and Guidance | (King et al., 2023) | It's a review. |
| 33 | Large Language Models in Biomedical and Health Informatics: A Review with Bibliometric Analysis | (Yu et al., 2024) | It's a review. |
| 34 | A conversational agent framework for mental health screening: design, implementation, and usability | (Boian et al., 2024) | It’s not mainly about LLMs. |
| 35 | Multi-layer Partial Information Fusion Model for Psychological Crisis Identification of Online Forum Users | (Liu et al., 2021) | It’s not mainly about LLMs. |
| 36 | Predicting implicit attitudes with natural language data | (Sudeep & Lukasz, 2023) | It’s not mainly about LLMs. |
| 37 | Post-traumatic Stress Disorder – Assessment of Current Diagnostic Definitions | (Bondjers, 2020) | It’s not mainly about LLMs. |
| 38 | Distinguishing clinical and genetic risk factors for suicidal ideation and behavior in a diverse hospital population | (Douglas et al., 2023) | It’s not mainly about LLMs. |
| 39 | Temporal communication dynamics in the aftermath of large-scale upheavals: do digital footprints reveal a stage model? | (Flores & Hilbert, 2023) | It’s not mainly about LLMs. |
| 40* | Exploring Mental Health Education for University Students in the Age of Large Language Models | (Liu & Li, 2023) | It’s not mainly about LLMs. |
| 41 | Predicting the language of depression from multivariate twitter data using a feature-rich hybrid deep learning model | (Kour & Gupta, 2022) | It’s not mainly about LLMs. |
| 42* | Research on Detection of the Causes of Suicidal | (Liu, 2020) | It’s not mainly about LLMs. |
| 43* | Enabling large-scale research on autism spectrum disorders | (Leroy, 2017) | It’s not mainly about LLMs. |
| 44* | Research on Textual Adversarial Attack Against Deep Learning Model | (Andy, 2021) | It’s not mainly about LLMs. |
| 45* | Predicting Self-Harm, Suicide Attempt, and Suicidal Death using Longitudinal EHR, Claims and Mortality Data | (George et al., 2019) | It’s not mainly about LLMs. |
| 46* | Identification of Trauma-related Features in EHR Data for Patients with Psychosis and Mood Disorders | (Hall et al., 2022) | It’s not mainly about LLMs. |
| 47 | Predicting and Characterizing the Health of Individuals and Communities through Language Analysis of Social Media | (Eichstaedt, 2017) | It’s not mainly about LLMs. |
| 48 | Natural language processing of clinical mental health notes may add predictive value to existing suicide risk models | (Levis et al., 2021) | It’s not mainly about LLMs. |
| 49 | Plan Explanations that Exploit a Cognitive Spatial Model | (Korpan et al., 2021) | It’s not mainly about LLMs. |
| 50 | Stress detection using natural language processing and machine learning over social interactions | (Nijhawan et al., 2022) | It’s not mainly about LLMs. |
| 51 | Detecting schizophrenia, bipolar disorder, psychosis vulnerability and major depressive disorder from 5 minutes of online-collected speech | (Olah et al., 2024) | It’s not mainly about LLMs. |
| 52 | Influencing human-AI interaction by priming beliefs about AI can increase perceived trustworthiness, empathy and effectiveness | (Pataranutaporn et al., 2023) | It’s not mainly about LLMs. |
| 53 | Can x2vec save lives? Integrating graph and language embeddings for automatic mental health classification | (Ruch, 2020) | It’s not mainly about LLMs. |
| 54 | CROSS-DEMOGRAPHIC PORTABILITY OF DEEP NLP-BASED DEPRESSION MODELS | (Rutowski et al., 2021) | It’s not mainly about LLMs. |
| 55 | What Can AI Do in Precision Psychiatry? A Study in Electronic Health Records | (Sheu, 2019) | It’s not mainly about LLMs. |
| 56 | Using natural language processing to identify opioid use disorder in electronic health record data | (Singleton et al., 2023) | It’s not mainly about LLMs. |
| 57 | Identifying emerging trends and hot topics through intelligent data mining: the case of clinical psychology and psychotherapy | (Sokolova et al., 2024) | It’s not mainly about LLMs. |
| 58 | Predicting Patients' Satisfaction With Mental Health Drug Treatment Using Their Reviews: Unified Interchangeable Model Fusion Approach | (Wang et al., 2023) | It’s not mainly about LLMs. |
| 59 | Efficient Reuse of Natural Language Processing Models for Phenotype-Mention Identification in Free-text Electronic Medical Records: A Phenotype Embedding Approach | (Wu et al., 2019) | It’s not mainly about LLMs. |
| 60 | Hierarchical Convolutional Attention Network for Depression Detection on Social Media and Its Impact During Pandemic | (Zogan et al., 2024) | It’s not mainly about LLMs. |
| 61 | Toward Automatic Tutoring of Math Word Problems in Intelligent Tutoring Systems | (Arnau-González et al., 2023) | It’s not mainly about LLMs. |
| 62 | REDE - Detecting human emotions using CNN and RASA | (Gupta et al., 2022) | It’s not mainly about LLMs. |
| 63 | Detection of multiple emotions in texts using a new deep convolutional neural network | (Izadkhah, 2022) | It’s not mainly about LLMs. |
| 64 | GUI: An Interface for Hate Speech Detection using NLP Technique | (Jain & Sharma, 2023) | It’s not mainly about LLMs. |
| 65 | Deep Learning and Natural Language Processing-Based Model for the Prediction of Suicidal Ideation in Military Personnel | (Katoch et al., 2023) | It’s not mainly about LLMs. |
| **66** | **Hybrid LSTM-TCN Model for Predicting Depression using Twitter Data** | **(Kour & Gupta, 2022)** | **It’s not mainly about LLMs.** |
| 67 | Does Social Media Feed Tell about Your Mental State? A Deep Randomised Neural Network Approach | (Kumar & Nisha 2022) | It’s not mainly about LLMs. |
| 68 | Novel Transformer Based Contextualized Embedding and Probabilistic Features for Depression Detection From Social Media | (Abbas et al., 2024) | It’s not mainly about LLMs. |
| 69 | News Media Framing of Suicide Circumstances and Gender: Mixed Methods Analysis | (Foriest et al., 2024) | It’s not mainly about LLMs. |
| 70 | A Multi-Class Deep Learning Approach for Early Detection of Depressive and Anxiety Disorders Using Twitter Data | (Bendebane et al., 2023) | It’s not mainly about LLMs. |
| 71 | Examining the role of AI technology in online mental healthcare: opportunities, challenges, and implications, a mixed-methods review | (Gutierrez et al., 2024) | It’s not mainly about LLMs. |
| 72 | Bored to death: Artificial Intelligence research reveals the role of boredom in suicide behavior | (Lissak et al., 2024) | It’s not mainly about LLMs. |
| 73 | Detecting Symptoms of Depression on Reddit | (Liu et al., 2023) | It’s not mainly about LLMs. |
| 74 | Assessing and managing the suicidal patient: forget the Reverend Bayes and try game theory | (Nielssen, 2024) | It’s not mainly about LLMs. |
| 75 | Predictive Analytics in Mental Health Leveraging LLM Embeddings and Machine Learning Models for Social Media Analysis | (Radwan et al., 2024) | It’s not mainly about LLMs. |
| 76 | Public Surveillance of Social Media for Suicide Using Advanced Deep Learning Models in Japan: Time Series Study From 2012 to 2022 | (Wang et al., 2023) | It’s not mainly about LLMs. |
| 77 | An Intelligent Assistant Diagnosis Method for Autistic Children based on Chinese Multimodal Discourse Corpus | (Liang, 2022) | It’s not mainly about LLMs. |
| 78 | Machine Feeling by Knowledge Acquisition with Emotion Map | (Lim et al., 2024) | It’s not mainly about LLMs. |
| 79 | An Intelligent Psychological Emotion Management System based on Multi-turn Voice Dialogue | (Lin et al., 2024) | It’s not mainly about LLMs. |
| 80 | Hybrid Model for Analysis of Social Media Posts for Identification of Depression and Measuring Its Severity | (Nanavati & Patel, 2023) | It’s not mainly about LLMs. |
| 81 | Design of Mental Health Consultation System Based on Deep Learning Algorithm | (Ran & Han, 2023) | It’s not mainly about LLMs. |
| 82 | Addressing the Productivity Paradox in Healthcare with Retrieval Augmented Generative AI Chatbots | (Ranasinghe et al., 2024) | It’s not mainly about LLMs. |
| 83 | Mental Health Disorder Identification From Motivational Conversations | (Saha et al., 2023) | It’s not mainly about LLMs. |
| 84 | Review on Early Prediction of Body Dysmorphic Disorder Using Machine Learning | (Saini & Prasad, 2023) | It’s not mainly about LLMs. |
| 85 | Daily Mental Health Monitoring from Speech: A Real-World Japanese Dataset and Multitask Learning Analysis | (Song et al., 2023) | It’s not mainly about LLMs. |
| 86 | User Feedback Severity Level Identification and Classification through Deeper Analysis of Text | (Umair et al., 2023) | It’s not mainly about LLMs. |
| 87 | A Multilevel Predictive Model for Detecting Social Network Users with Depression | (Wongkoblap et al., 2018) | It’s not mainly about LLMs. |
| 88 | HICEM: A High-Coverage Emotion Model for Artificial Emotional Intelligence | (Wortman & Wang, 2024) | It’s not mainly about LLMs. |
| 89 | Climate and Weather: Inspecting Depression Detection via Emotion Recognition | (Wu et al., 2022) | It’s not mainly about LLMs. |
| 90 | Design of Intelligent Dispatching System Based on Human Voice Adaptive Speech Recognition | (Xiang et al., 2021) | It’s not mainly about LLMs. |
| 91 | Assessing dimensions of thought disorder with large language models: The tradeoff of accuracy and consistency | (Pugh et al., 2024) | It’s not mainly about LLMs. |
| 92 | Natural Language Processing for Depression Prediction on Sina Weibo: Method Study and Analysis | (Zhang et al., 2024) | It’s not mainly about LLMs. |
| 93 | Identifying Suicidal Adolescents from Mental Health Records Using Natural Language Processing | (Velupillai et al., 2019) | It’s not mainly about LLMs. |
| 94 | Understanding the Benefits and Challenges of Using Large Language Model-based Conversational Agents for Mental Well-being Support | (Ma et al., 2023) | It has not yet been published in a journal or at a conference. |
| 95 | Visualizing Mental Health Insights: A Pipeline from Social Media to Chernoff Faces | (Nagi et al., 2024) | It has not yet been published in a journal or at a conference. |
| 96 | Clinical decision support for bipolar depression using large language models | (Perlis et al., 2024) | It has not yet been published in a journal or at a conference. |
| 97* | Computational ontology of brain systems across the human neuroimaging literature | (Beam et al., 2021) | It has not yet been published in a journal or at a conference. |
| 98* | Research on Text Sentiment Analysis Based on Deep Pretraining Language Model | (He, 2021) | It has not yet been published in a journal or at a conference. |
| 99* | Research on Key Technologies for Social Media Text Sentiment Analysis | (Li, 2020) | It has not yet been published in a journal or at a conference. |
| 100* | Research and Implementation of DomainOriented Text Sentiment Analysis Technology Based on Deep Learnin | (Chen, 2019) | It has not yet been published in a journal or at a conference. |
| 101* | Depression Detection Research Based on Social Media | (Cui, 2023) | It has not yet been published in a journal or at a conference. |
| 102* | Design and Implementation of the Software System of Psychological Consultation Robot Based on Deep Learning | (Yan, 2023) | It has not yet been published in a journal or at a conference. |
| 103 | Towards Clinically Improved NLP for Psycholinguistic Understanding | (Aich, 2024) | It has not yet been published in a journal or at a conference. |
| 104 | Exploring Large Language Models for Mental Health Analysis: Performance Evaluation and Comprehensive Analysis on Different Reddit Mental Health Communities | (Bhandari, 2024) | It has not yet been published in a journal or at a conference. |
| 105 | Empowering Emotional Support Chatbots With Large Language Models | (Pushparaj, 2024) | It has not yet been published in a journal or at a conference. |
| 106 | Neonatal Toxic Stress and Long-Term Neurodevelopment in Premature Infants | (Sey, 2021) | It has not yet been published in a journal or at a conference. |
| 107 | Human-AI Collaboration to Support Mental Health and Well-Being | (Sharma, 2024) | It has not yet been published in a journal or at a conference. |
| 108 | Harnessing Large Language Models for Mental Health: From Sentiment Analysis to Depression Screening | (Sood, 2024) | It has not yet been published in a journal or at a conference. |
| 109 | Essays on Digital Technology-Enabled Mental Healthcare Delivery | (Tang, 2024) | It has not yet been published in a journal or at a conference. |
| 110* | Harnessing Large Language Models to measure and improve autobiographical memory problems among people with mental health problems | (Tom & Hallford, 2024) | It has not yet been published in a journal or at a conference. |
| 111 | Detecting Risky Alcohol Use With Natural Language Processing and Computable Phenotypes in Clinical Records | (Weber, 2024) | It has not yet been published in a journal or at a conference. |
| 112 | Investigating Major Topics Through the Analysis of Depression-related Facebook Group Posts | (Zhu et al. 2019) | It has not yet been published in a journal or at a conference. |
| 113* | Prostate cancer is a heterogeneous disease, displaying a multitude of genetic alterations, histological patterns and clinical outcomes. This heterogen | (Nevado-Holgado and Taylor, 2024) | It has not yet been published in a journal or at a conference. |
| 114* | Unlocking mental health records at scale using few-shot AI | (Gao, 2024) | It has not yet been published in a journal or at a conference. |
| 115 | Speech and Text Psychometrics: Identifying Suicide Risk Factors With Large Language Models and Acoustic Networks | (Low, 2024) | It has not yet been published in a journal or at a conference. |
| 116* | Leveraging Large Language Models and Machine Learning Algorithms to Assess Depression and Anxiety Symptoms and Risks for Patients with Cardiovascular Disease or Diabetes Mellitus | (Kim, 2024) | It has not yet been published in a journal or at a conference. |
| 117 | Textual Mental Illness Detection from Social Media Using Deep Learning | (Zhang, 2024) | It has not yet been published in a journal or at a conference. |
| 118 | [Performance of generative pre-trained transformer-4 on the certification test for mental health management: A factorial design] | (Watanabe et al., 2024) | It has not yet been published in a journal or at a conference. |
| 119 | Mental Health Prediction from Social Media Text Using Mixture of Experts | (dos Santos et al., 2023) | It has not yet been published in a journal or at a conference. |
| 120 | Mental-Health Topic Classification employing D-vectors of Large Language Models | (Luna-Jimenéz et al., 2024) | It has not yet been published in a journal or at a conference. |
| 121 | Gaze and Head Movement Patterns of Depressive Symptoms During Conversations with Emotional Virtual Humans | (Marín-Morales, Llanes-Jurado et al. 2023) | It has not yet been published in a journal or at a conference. |
| 122 | Conceptualizing Suicidal Behavior: Utilizing Explanations of Predicted Outcomes to Analyze Longitudinal Social Media Data | (Marín-Morales et al., 2023) | It has not yet been published in a journal or at a conference. |
| 123 | ProDepDet: Out-of-domain Knowledge Transfer of Pre-trained Large Language Models for Depression Detection in Text-Based Multi-Party Conversations | (Priyadarshana et al., 2024) | It has not yet been published in a journal or at a conference. |
| 124 | Comparing Conventional Machine Learning and Large-Language Models for Human Stress Detection Using Social Media Posts | (Ramteke and Khandelwal, 2023) | It has not yet been published in a journal or at a conference. |
| 125 | A Machine Learning Enabled Approach for Mental and Physical Health Management Using OpenCV, NLP and IOT | (Rane, Khanke et al. 2024) | It has not yet been published in a journal or at a conference. |
| 126 | Transformer Models for Recognizing Abusive Language An investigation and review on Tweeteval and SOLID dataset | (Rawther & Titus, 2023) | It has not yet been published in a journal or at a conference. |
| 127 | Analyzing the Performance of Machine Learning and Deep Learning Models in Detecting Cyberbullying Comments | (Saim et al., 2023) | It has not yet been published in a journal or at a conference. |
| 128 | RAG-Based LLM Chatbot Using Llama-2 | (Vakayil et al., 2024) | It has not yet been published in a journal or at a conference. |
| 129 | AI-Enhanced Mental Health Diagnosis: Leveraging Transformers for Early Detection of Depression Tendency in Textual Data | (Verma et al., 2023) | It has not yet been published in a journal or at a conference. |
| 130 | Investigating Large Language Models’ Perception of Emotion Using Appraisal Theory | (Yongsatianchot et al., 2023) | It has not yet been published in a journal or at a conference. |
| 131 | Development of Serious Game Theory Framework in Virtual Reality for Alzheimer's Patients | (Zuo et al., 2024) | It has not yet been published in a journal or at a conference. |
| 132 | A Therapeutic Dialogue Agent for Polish Language | (Zygadlo, 2021) | It has not yet been published in a journal or at a conference. |

Table S4. List of studies excluded at the full-text screening stage.

*: These articles do not contain verifiable references, including awards, dissertations, research projects etc.

References

Abbas, M. A., Munir, K., Raza, A., Samee, N. A., Jamjoom, M. M., & Ullah, Z. (2024). Novel transformer based contextualized embedding and probabilistic features for depression detection from social media. IEEE Access.

Aich, A. (2024). Towards Clinically Improved NLP for Psycholinguistic Understanding (Doctoral dissertation, University of Illinois at Chicago).

Alam, M. A. U., & Kapadia, D. (2020, September). Laxary: a trustworthy explainable twitter analysis model for post-traumatic stress disorder assessment. In 2020 IEEE International Conference on Smart Computing (SMARTCOMP) (pp. 308-313). IEEE.

Arnau-González, P., Serrano-Mamolar, A., Katsigiannis, S., Althobaiti, T., & Arevalillo-Herráez, M. (2023). Toward automatic tutoring of math word problems in intelligent tutoring systems. IEEE Access, 11, 67030-67039.

Bendebane, L., Laboudi, Z., Saighi, A., Al-Tarawneh, H., Ouannas, A., & Grassi, G. (2023). A Multi-class deep learning approach for early detection of depressive and anxiety disorders using Twitter data. Algorithms, 16(12), 543.

Berrezueta-Guzman, S., Kandil, M., Martín-Ruiz, M. L., de la Cruz, I. P., & Krusche, S. (2024, June). Exploring the efficacy of robotic assistants with ChatGPT and Claude in enhancing ADHD therapy: Innovating treatment paradigms. In 2024 International Conference on Intelligent Environments (IE) (pp. 25-32). IEEE.

Bhandari, N. (2024). Exploring Large Language Models for Mental Health Analysis: Performance Evaluation and Comprehensive Analysis on Different Reddit Mental Health Communities (Master's thesis, Lamar University-Beaumont).

Bhatia, S., & Walasek, L. (2023). Predicting implicit attitudes with natural language data. Proceedings of the National Academy of Sciences, 120(25), e2220726120.

Blease, C., & Torous, J. (2023). ChatGPT and mental healthcare: balancing benefits with risks of harms. BMJ Ment Health, 26(1).

Boian, R., Bucur, A. M., Todea, D., Luca, A., Rebedea, T., & Podina, I. R. (2024). A conversational agent framework for mental health screening: Design, implementation, and usability. Behaviour & Information Technology, 1-15.

Bondjers, K. (2020). Post-traumatic stress disorder–Assessment of current diagnostic definitions (Doctoral dissertation, Acta Universitatis Upsaliensis).

Cho, Y. M., Rai, S., Ungar, L., Sedoc, J., & Guntuku, S. C. (2023, December). An integrative survey on mental health conversational agents to bridge computer science and medical perspectives. In Proceedings of the Conference on Empirical Methods in Natural Language Processing. Conference on Empirical Methods in Natural Language Processing (Vol. 2023, p. 11346).

Colbert, S. M. C., Lepow, L., Fennessy, B., Iwata, N., Ikeda, M., Saito, T., Terao, C., Preuss, M., Pathak, J., Mann, J. J., Coon, H., & Mullins, N. (2025). Distinguishing clinical and genetic risk factors for suicidal ideation and behavior in a diverse hospital population. Translational psychiatry, 15(1), 63. https://doi.org/10.1038/s41398-025-03287-6

De-xi, L. I. U., Li-ping, B. A. O., Chang-xuan, W. A. N., Xi-ping, L. I. U., & Guo-qiong, L. I. A. O. (2021). Multi-layer partial information fusion model for psychological crisis identification of online forum users. Journal of Chinese Computer Systems, 42(4), 690-699.

Eichstaedt, J. C. (2017). Predicting and characterizing the health of individuals and communities through language analysis of social media. University of Pennsylvania.

Elyoseph, Z., & Levkovich, I. (2024). Comparing the perspectives of generative AI, mental health experts, and the general public on schizophrenia recovery: case vignette study. JMIR Mental Health, 11, e53043.

Feng, Y., Xu, X., Zhuang, Y., & Zhang, M. (2023, November). Large language models improve Alzheimer's disease diagnosis using multi-modality data. In 2023 IEEE International Conference on Medical Artificial Intelligence (MedAI) (pp. 61-66). IEEE.

Ferrario, A., Sedlakova, J., & Trachsel, M. (2024). The role of humanization and robustness of large language models in conversational artificial intelligence for individuals with depression: a critical analysis. JMIR Mental Health, 11, e56569.

Flores, P. M., & Hilbert, M. (2023). Temporal communication dynamics in the aftermath of large-scale upheavals: do digital footprints reveal a stage model?. Journal of Computational Social Science, 6(2), 973-999.

Foriest, J. C., Mittal, S., Kim, E., Carmichael, A., Lennon, N., Sumner, S. A., & De Choudhury, M. (2024). News media framing of suicide circumstances and gender: mixed methods analysis. JMIR mental health, 11(1), e49879.

Ganesan, A. V., Matero, M., Ravula, A. R., Vu, H., & Schwartz, H. A. (2021, June). Empirical evaluation of pre-trained transformers for human-level NLP: The role of sample size and dimensionality. In Proceedings of the conference. Association for Computational Linguistics. North American Chapter. Meeting (Vol. 2021, p. 4515).

García-Méndez, S., & de Arriba-Pérez, F. (2024). Large language models and healthcare alliance: potential and challenges of two representative use cases. Annals of Biomedical Engineering, 52(8), 1928-1931.

Gupta, A., Raj, M. A., Singh, K., & Deshmukh, R. (2022, January). REDE-Detecting human emotions using CNN and RASA. In 2022 International Conference for Advancement in Technology (ICONAT) (pp. 1-6). IEEE.

Gutierrez, G., Stephenson, C., Eadie, J., Asadpour, K., & Alavi, N. (2024). Examining the role of AI technology in online mental healthcare: opportunities, challenges, and implications, a mixed-methods review. Frontiers in psychiatry, 15, 1356773.

Haber, Y., Levkovich, I., Hadar-Shoval, D., & Elyoseph, Z. (2024). The artificial third: a broad view of the effects of introducing generative artificial intelligence on psychotherapy. JMIR Mental Health, 11, e54781.

Han, G., Liu, W., Huang, X., & Borsari, B. (2024, June). Chain-of-interaction: Enhancing large language models for psychiatric behavior understanding by dyadic contexts. In 2024 IEEE 12th International Conference on Healthcare Informatics (ICHI) (pp. 392-401). IEEE.

Hasan, M., Ozel, C., Potter, S., & Hoque, E. (2023, September). SAPIEN: affective virtual agents powered by large language models. In 2023 11th International Conference on Affective Computing and Intelligent Interaction Workshops and Demos (ACIIW) (pp. 1-3). IEEE.

Hodson, N., & Williamson, S. (2024). Can large language models replace therapists? Evaluating performance at simple cognitive behavioral therapy tasks. JMIR AI, 3(1), e52500.

Izadkhah, H. (2022, March). Detection of multiple emotions in texts using a new deep convolutional neural network. In 2022 9th Iranian Joint Congress on Fuzzy and Intelligent Systems (CFIS) (pp. 1-6). IEEE.

Jain, A., & Sharma, S. (2023, August). GUI: An Interface for Hate Speech Detection using NLP Technique. In 2023 Second International Conference on Augmented Intelligence and Sustainable Systems (ICAISS) (pp. 1144-1148). IEEE.

Katoch, S., Dhaliwal, B. K., & Singh, G. (2023, March). Deep Learning and Natural Language Processing-Based Model for the Prediction of Suicidal Ideation in Military Personnel. In 2023 10th International Conference on Signal Processing and Integrated Networks (SPIN) (pp. 508-514). IEEE.

King, D. R., Nanda, G., Stoddard, J., Dempsey, A., Hergert, S., Shore, J. H., & Torous, J. (2023). An introduction to generative artificial intelligence in mental health care: considerations and guidance. Current psychiatry reports, 25(12), 839-846.

Kiuchi, K., Otsu, K., & Hayashi, Y. (2024). Psychological insights into the research and practice of embodied conversational agents, chatbots and social assistive robots: a systematic meta-review. Behaviour & Information Technology, 43(15), 3696-3736.

Kjell, O. N., Kjell, K., & Schwartz, H. A. (2024). Beyond rating scales: With targeted evaluation, large language models are poised for psychological assessment. Psychiatry Research, 333, 115667.

Korpan, R., & Epstein, S. L. (2021, August). Plan Explanations that Exploit a Cognitive Spatial Model. In Proceedings of Second International Combined Workshop on Spatial Language Understanding and Grounded Communication for Robotics (pp. 60-70).

Kour, H., & Gupta, M. K. (2022). Predicting the language of depression from multivariate twitter data using a feature‐rich hybrid deep learning model. Concurrency and Computation: Practice and Experience, 34(24), e7224.

Kour, H., & Gupta, M. K. (2022, December). Hybrid lstm-tcn model for predicting depression using twitter data. In 2022 17th International Conference on Control, Automation, Robotics and Vision (ICARCV) (pp. 167-172). IEEE.

Kumar, S., & Nisha, Z. (2022, July). Does social media feed tell about your mental state? A deep randomised neural network approach. In 2022 international joint conference on neural networks (IJCNN) (pp. 1-8). IEEE.

Lawrence, H. R., Schneider, R. A., Rubin, S. B., Matarić, M. J., McDuff, D. J., & Bell, M. J. (2024). The opportunities and risks of large language models in mental health. JMIR Mental Health, 11(1), e59479.

Levis, M., Westgate, C. L., Gui, J., Watts, B. V., & Shiner, B. (2021). Natural language processing of clinical mental health notes may add predictive value to existing suicide risk models. Psychological medicine, 51(8), 1382-1391.

Liang, Y. (2022, December). An Intelligent Assistant Diagnosis Method for Autistic Children based on Chinese Multimodal Discourse Corpus. In 2022 2nd International Conference on Networking, Communications and Information Technology (NetCIT) (pp. 537-541). IEEE.

Lim, C. G., Choi, H. J., Mswahili, M. E., Ndomba, G. E., & Jeong, Y. S. (2024, February). Machine Feeling by Knowledge Acquisition with Emotion Map. In 2024 IEEE International Conference on Big Data and Smart Computing (BigComp) (pp. 90-96). IEEE.

Lin, N., Lin, Y., & Xu, Y. (2024, March). An Intelligent Psychological Emotion Management System based on Multi-turn Voice Dialogue. In 2024 5th International Seminar on Artificial Intelligence, Networking and Information Technology (AINIT) (pp. 461-465). IEEE.

Lissak, S., Ophir, Y., Tikochinski, R., Brunstein Klomek, A., Sisso, I., Fruchter, E., & Reichart, R. (2024). Bored to death: Artificial Intelligence research reveals the role of boredom in suicide behavior. Frontiers in psychiatry, 15, 1328122.

Liu, T., Jain, D., Rapole, S. R., Curtis, B., Eichstaedt, J. C., Ungar, L. H., & Guntuku, S. C. (2023, April). Detecting symptoms of depression on reddit. In Proceedings of the 15th ACM web science conference 2023 (pp. 174-183).

Low, D. M. (2024). Speech and text psychometrics: Identifying suicide risk factors with large language models and acoustic networks. Harvard University.

Luna-Jimenéz, C., Callejas, Z., & Griol, D. (2024, June). Mental-Health Topic Classification employing D-vectors of Large Language Models. In 2024 IEEE 37th International Symposium on Computer-Based Medical Systems (CBMS) (pp. 199-204). IEEE.

Lundin, R. M., Berk, M., & Østergaard, S. D. (2023). ChatGPT on ECT: can large language models support psychoeducation?. The journal of ECT, 39(3), 130-133.

Ma, Z., Mei, Y., & Su, Z. (2024, January). Understanding the benefits and challenges of using large language model-based conversational agents for mental well-being support. In AMIA Annual Symposium Proceedings (Vol. 2023, p. 1105).

Marín-Morales, J., Llanes-Jurado, J., Minissi, M. E., Gómez-Zaragozá, L., Altozano, A., & Alcañiz, M. (2023, September). Gaze and head movement patterns of depressive symptoms during conversations with emotional virtual humans. In 2023 11th International Conference on Affective Computing and Intelligent Interaction (ACII) (pp. 1-8). IEEE.

Mei, K., Fereidooni, S., & Caliskan, A. (2023, June). Bias against 93 stigmatized groups in masked language models and downstream sentiment classification tasks. In Proceedings of the 2023 ACM Conference on Fairness, Accountability, and Transparency (pp. 1699-1710).

Mostafazadeh, N., Kalyanpur, A., Moon, L., Buchanan, D., Berkowitz, L., Biran, O., & Chu-Carroll, J. (2020). GLUCOSE: GeneraLized and COntextualized story explanations. arXiv preprint arXiv:2009.07758.

Nagi, F., Alzubaidi, M., Shah, U., Shah, H., Alabdulla, M., Househ, M., & Agus, M. (2024). Visualizing Mental Health Insights: A Pipeline from Social Media to Chernoff Faces. In Digital Health and Informatics Innovations for Sustainable Health Care Systems (pp. 1972-1976). IOS Press.

Nanavati, J., & Patel, U. (2023, July). Hybrid Model for Analysis of Social Media Posts for Identification of Depression and Measuring Its Severity. In 2023 International Conference on Data Science and Network Security (ICDSNS) (pp. 1-5). IEEE.

Nielssen, O. (2024). Assessing and managing the suicidal patient: forget the Reverend Bayes and try game theory. BJPsych Bulletin, 1-5.

Nijhawan, T., Attigeri, G. & Ananthakrishna, T. Stress detection using natural language processing and machine learning over social interactions. J Big Data 9, 33 (2022). https://doi.org/10.1186/s40537-022-00575-6

Nur, N., Stern, W., Mercer, T., Sen, C., Bhattacharyya, S., Tumbiolo, V., & Goh, S. J. (2023, December). Conceptualizing Suicidal Behavior: Utilizing Explanations of Predicted Outcomes to Analyze Longitudinal Social Media Data. In 2023 International Conference on Machine Learning and Applications (ICMLA) (pp. 2095-2102). IEEE.

Olah, J., Wong, W. L. E., Chaudhry, A. U. R. R., Mena, O., & Tang, S. X. (2024). Detecting schizophrenia, bipolar disorder, psychosis vulnerability and major depressive disorder from 5 minutes of online-collected speech. medRxiv.

Pataranutaporn, P., Liu, R., Finn, E., & Maes, P. (2023). Influencing human–AI interaction by priming beliefs about AI can increase perceived trustworthiness, empathy and effectiveness. Nature Machine Intelligence, 5(10), 1076-1086.

Perlis, R. H., Goldberg, J. F., Ostacher, M. J., & Schneck, C. D. (2024). Clinical decision support for bipolar depression using large language models. Neuropsychopharmacology, 49(9), 1412-1416.

Priyadarshana, Y. H. P. P., Liang, Z., & Piumarta, I. (2024, June). ProDepDet: Out-of-domain Knowledge Transfer of Pre-trained Large Language Models for Depression Detection in Text-Based Multi-Party Conversations. In 2024 International Joint Conference on Neural Networks (IJCNN) (pp. 1-8). IEEE.

Priyadarshana, Y. H. P. P., Senanayake, A., Liang, Z., & Piumarta, I. (2024). Prompt engineering for digital mental health: a short review. Frontiers in Digital Health, 6, 1410947.

Pugh, S. L., Chandler, C., Cohen, A. S., Diaz-Asper, C., Elvevåg, B., & Foltz, P. W. (2024). Assessing dimensions of thought disorder with large language models: The tradeoff of accuracy and consistency. Psychiatry Research, 341, 116119.

Pushparaj, S. R. R. (2024). Empowering Emotional Support Chatbots with Large Language Models (Master's thesis, State University of New York at Buffalo).

Radwan, A., Amarneh, M., Alawneh, H., Ashqar, H. I., AlSobeh, A., & Magableh, A. A. A. R. (2024). Predictive analytics in mental health leveraging LLM embeddings and machine learning models for social media analysis. International Journal of Web Services Research (IJWSR), 21(1), 1-22.

Raile, P. (2024). The usefulness of ChatGPT for psychotherapists and patients. Humanities and Social Sciences Communications, 11(1), 1-8.

Ramteke, P. S., & Khandelwal, S. (2023, November). Comparing Conventional Machine Learning and Large-Language Models for Human Stress Detection Using Social Media Posts. In 2023 2nd International Conference on Futuristic Technologies (INCOFT) (pp. 1-8). IEEE.

Ran, Q., & Han, D. (2023, October). Design of Mental Health Consultation System Based on Deep Learning Algorithm. In 2023 International Conference on Computer Science and Automation Technology (CSAT) (pp. 257-262). IEEE.

Ranasinghe, S., De Silva, D., Mills, N., Alahakoon, D., Manic, M., Lim, Y., & Ranasinghe, W. (2024, March). Addressing the productivity paradox in healthcare with retrieval augmented generative AI chatbots. In 2024 IEEE International Conference on Industrial Technology (ICIT) (pp. 1-6). IEEE.

Rane, M., Khanke, R., Kharat, K., Kolhe, K., Mane, R., & Vaidya, J. (2024, March). A Machine Learning Enabled Approach for Mental and Physical Health Management Using OpenCV, NLP and IOT. In 2024 International Conference on Emerging Smart Computing and Informatics (ESCI) (pp. 1-8). IEEE.

Rawther, F. A., & Titus, G. (2023, April). Transformer Models for Recognizing Abusive Language An investigation and review on Tweeteval and SOLID dataset. In 2023 Second International Conference on Electrical, Electronics, Information and Communication Technologies (ICEEICT) (pp. 1-6). IEEE.

Ruch, A. (2020). Can x2vec save lives? integrating graph and language embeddings for automatic mental health classification. Journal of Physics: Complexity, 1(3), 035005.

Rutowski, T., Shriberg, E., Harati, A., Lu, Y., Oliveira, R., & Chlebek, P. (2021, January). Cross-demographic portability of deep NLP-based depression models. In 2021 IEEE spoken language technology workshop (SLT) (pp. 1052-1057). IEEE.

Saha, T., Reddy, S. M., Saha, S., & Bhattacharyya, P. (2022). Mental health disorder identification from motivational conversations. IEEE Transactions on Computational Social Systems, 10(3), 1130-1139.

Saim, M., Rizvi, R. M., & Khan, M. K. (2023, November). Analyzing the Performance of Machine Learning and Deep Learning Models in Detecting Cyberbullying Comments. In 2023 International Conference on Recent Advances in Science and Engineering Technology (ICRASET) (pp. 1-6). IEEE.

Saini, H., & Prasad, S. K. (2023, August). Review on Early Prediction of Body Dysmorphic Disorder Using Machine Learning. In 2023 Second International Conference On Smart Technologies For Smart Nation (SmartTechCon) (pp. 1140-1146). IEEE.

Santos, W., Yoon, S., & Paraboni, I. (2023). Mental health prediction from social media text using mixture of experts. IEEE Latin America Transactions, 21(6), 723-729.

Sey, R. R. (2021). Neonatal Toxic Stress and Long-Term Neurodevelopment in Premature Infants. University of San Diego.

Sezgin, E., Chekeni, F., Lee, J., & Keim, S. (2023). Clinical accuracy of large language models and Google search responses to postpartum depression questions: cross-sectional study. Journal of Medical Internet Research, 25, e49240.

Sharma, A. (2024). Human-AI Collaboration to Support Mental Health and Well-Being (Doctoral dissertation, University of Washington).

Sharma, A., Rushton, K., Lin, I. W., Wadden, D., Lucas, K. G., Miner, A. S., ... & Althoff, T. (2023). Cognitive reframing of negative thoughts through human-language model interaction. arXiv preprint arXiv:2305.02466.

Sheu, Y. H. (2019). What Can AI Do in Precision Psychiatry? A Study in Electronic Health Records. Harvard University.

Singleton, J., Li, C., Akpunonu, P. D., Abner, E. L., & Kucharska-Newton, A. M. (2023). Using natural language processing to identify opioid use disorder in electronic health record data. International Journal of Medical Informatics, 170, 104963.

Sokolova, A., Lobanova, P., & Kuzminov, I. (2024). Identifying emerging trends and hot topics through intelligent data mining: the case of clinical psychology and psychotherapy. foresight, 26(1), 155-180.

Song, M., Triantafyllopoulos, A., Yang, Z., Takeuchi, H., Nakamura, T., Kishi, A., ... & Yamamoto, Y. (2023, June). Daily mental health monitoring from speech: A real-world japanese dataset and multitask learning analysis. In ICASSP 2023-2023 IEEE International Conference on Acoustics, Speech and Signal Processing (ICASSP) (pp. 1-5). IEEE.

Sood, P. (2024). Harnessing Large Language Models for Mental Health: From Sentiment Analysis to Depression Screening (Master's thesis, Stevens Institute of Technology).

Stade, E. C., Stirman, S. W., Ungar, L. H., Boland, C. L., Schwartz, H. A., Yaden, D. B., ... & Eichstaedt, J. C. (2024). Large language models could change the future of behavioral healthcare: a proposal for responsible development and evaluation. NPJ Mental Health Research, 3(1), 12.

Sun, H., Lin, Z., Zheng, C., Liu, S., & Huang, M. (2021). Psyqa: A chinese dataset for generating long counseling text for mental health support. arXiv preprint arXiv:2106.01702.

Tanana, M. J., Soma, C. S., Kuo, P. B., Bertagnolli, N. M., Dembe, A., Pace, B. T., ... & Imel, Z. E. (2021). How do you feel? Using natural language processing to automatically rate emotion in psychotherapy. Behavior research methods, 1-14.

Tang, Y. (2024). Essays on Digital Technology-Enabled Mental Healthcare Delivery (Doctoral dissertation, University of Minnesota).

Tao, J., Fan, C., Lian, Z., Lyu, Z., Shen, Y., & Liang, S. (2024). Development of multimodal sentiment recognition and understanding. Journal of Image and Graphics.

Umair, M., Irtaza, S. A., & Salim, S. (2023, March). User Feedback Severity Level Identification and Classification through Deeper Analysis of Text. In 2023 4th International Conference on Computing, Mathematics and Engineering Technologies (iCoMET) (pp. 1-7). IEEE.

Vakayil, S., Juliet, D. S., & Vakayil, S. (2024, April). RAG-Based LLM Chatbot Using Llama-2. In 2024 7th International Conference on Devices, Circuits and Systems (ICDCS) (pp. 1-5). IEEE.

Velupillai, S., Epstein, S., Bittar, A., Stephenson, T., Dutta, R., & Downs, J. (2019). Identifying suicidal adolescents from mental health records using natural language processing. In MEDINFO 2019: Health and Wellbeing e-Networks for All (pp. 413-417). IOS Press.

Verma, S., Joshi, R. C., Dutta, M. K., Jezek, S., & Burget, R. (2023, October). AI-enhanced mental health diagnosis: leveraging transformers for early detection of depression tendency in textual data. In 2023 15th International Congress on Ultra Modern Telecommunications and Control Systems and Workshops (ICUMT) (pp. 56-61). IEEE.

Volkmer, S., Meyer-Lindenberg, A., & Schwarz, E. (2024). Large language models in psychiatry: opportunities and challenges. Psychiatry research, 116026.

Wang, S., Ning, H., Huang, X., Xiao, Y., Zhang, M., Yang, E. F., ... & Zeng, Y. (2023). Public surveillance of social media for suicide using advanced deep learning models in Japan: time series study from 2012 to 2022. Journal of medical internet research, 25, e47225.

Wang, Y., Yu, Y., Liu, Y., Ma, Y., & Pang, P. C. I. (2023). Predicting Patients' satisfaction with mental health drug treatment using their reviews: unified interchangeable model fusion approach. JMIR Mental Health, 10(1), e49894.

Watanabe, K., Tsutsui, Y., Tsutsui, T., Yamauchi, T., Uchida, M., Hachiya, Y., ... & Kawakami, N. (2024). Performance of generative pre-trained transformer-4 on the certification test for mental health management: A factorial design. Sangyo eiseigaku zasshi= Journal of occupational health, 66(6), 303-313.

Weber, K. (2024). Detecting Risky Alcohol Use with Natural Language Processing and Computable Phenotypes in Clinical Records (Doctoral dissertation).

Wei, Y., Guo, L., Lian, C., & Chen, J. (2023). ChatGPT: opportunities, risks and priorities for psychiatry. Asian journal of psychiatry, 90, 103808.

Wongkoblap, A., Vadillo, M. A., & Curcin, V. (2018, June). A multilevel predictive model for detecting social network users with depression. In 2018 IEEE International Conference on Healthcare Informatics (ICHI) (pp. 130-135). IEEE.

Wortman, B., & Wang, J. Z. (2023). HICEM: A high-coverage emotion model for artificial emotional intelligence. IEEE Transactions on Affective Computing, 15(3), 1136-1152.

Wu, H., Hodgson, K., Dyson, S., Morley, K. I., Ibrahim, Z. M., Iqbal, E., ... & Sudlow, C. (2019). Efficient reuse of natural language processing models for phenotype-mention identification in free-text electronic medical records: a phenotype embedding approach. JMIR Medical Informatics, 7(4), e14782.

Wu, W., Wu, M., & Yu, K. (2022, May). Climate and weather: Inspecting depression detection via emotion recognition. In ICASSP 2022-2022 IEEE International Conference on Acoustics, Speech and Signal Processing (ICASSP) (pp. 6262-6266). IEEE.

Xiang, Z., Gu, W., Tong, C., Qian, X., Li, Z., & Guo, C. (2021, December). Design of Intelligent Dispatching System Based on Human Voice Adaptive Speech Recognition. In 2021 International Conference on Power System Technology (POWERCON) (pp. 1953-1957). IEEE.

Yongsatianchot, N., Torshizi, P. G., & Marsella, S. (2023, September). Investigating large language models’ perception of emotion using appraisal theory. In 2023 11th International Conference on Affective Computing and Intelligent Interaction Workshops and Demos (ACIIW) (pp. 1-8). IEEE.

Yu, H., Fan, L., Li, L., Zhou, J., Ma, Z., Xian, L., ... & Ma, X. (2024). Large language models in biomedical and health informatics: A review with bibliometric analysis. Journal of Healthcare Informatics Research, 8(4), 658-711.

Zhang, T. (2024). Textual Mental Illness Detection from Social Media Using Deep Learning (Doctoral dissertation, The University of Manchester (United Kingdom)).

Zhang, Y. F., & Liu, X. Q. (2024). Using ChatGPT to promote college students’ participation in physical activities and its effect on mental health. World Journal of Psychiatry, 14(2), 330.

Zhang, Z., Zhu, J., Guo, Z., Zhang, Y., Li, Z., & Hu, B. (2024). Natural Language Processing for Depression Prediction on Sina Weibo: Method Study and Analysis. JMIR Mental Health, 11, e58259.

Zhu, H., Neubig, G., & Bisk, Y. (2021, July). Few-shot language coordination by modeling theory of mind. In International Conference on Machine Learning (pp. 12901-12911). PMLR.

Zogan, H., Razzak, I., Jameel, S., & Xu, G. (2023). Hierarchical convolutional attention network for depression detection on social media and its impact during pandemic. IEEE Journal of Biomedical and Health Informatics, 28(4), 1815-1823.

Zuo, H., Lim, K. H., Eswaran, S., & Lease, B. A. (2024, January). Development of Serious Game Theory Framework in Virtual Reality for Alzheimer's Patients. In 2024 International Conference on Green Energy, Computing and Sustainable Technology (GECOST) (pp. 190-194). IEEE.

Zygadlo, A. (2021, September). A therapeutic dialogue agent for Polish language. In 2021 9th International Conference on Affective Computing and Intelligent Interaction Workshops and Demos (ACIIW) (pp. 1-5). IEEE.

주영준, 김동훈, 이창호, & 이용정. (2019). Investigating major topics through the analysis of depression-related facebook group posts. 한국문헌정보학회지, 53(4), 171-187.
