# Supplementary material for: The Applications of Large Language Models in Mental Health: Scoping Review
Source: J Med Internet Res. 2025 May 5;27:e69284. doi: 10.2196/69284 (PMC12089884; doi:10.2196/69284)
Supplement: Multimedia Appendix 6 [file jmir_v27i1e69284_app6.docx]

Supplemental Files

Table S5: Categorization of articles based on mental health applications across 95 studies.

| Applications | | Datasets | No | F1 score | Precision | ACC | Recall | AUC | Sensitivity | Specificity |
| --- | --- | --- | --- | --- | --- | --- | --- | --- | --- | --- |
| The screening, identification, or detection of mental disorders | Depression detection and classification | Reddit, Twitter, DAIC-WOZ, E-DAIC, Facebook, Weibo, DTR, Dreaddit, GoEmotions, etc. | 33 (35%) | 24 | 18 | 14 | 16 | 2 | 2 | 2 |
|  | Suicide risk prediction |  | 12 (13%) | 7 | 4 | 7 | 4 | 1 | 1 | 1 |
|  | Sentiment analysis |  | 9 (10%) | 6 | 4 | 7 | 4 | 0 | 0 | 0 |
|  | Others |  | 13 (14%) | 5 | 4 | 8 | 4 | 0 | 0 | 0 |
| Supporting the clinical treatments and interventions | Supporting the clinical treatments and interventions | DAIC-WOZ, E-DAIC, Weibo, Zhihu, Wikipedia, Encyclopedia, MedDialog, USPTO, etc. | 14 (15%) | 5 | 3 | 2 | 3 | 3 | 2 | 1 |
|  | Developing conversational virtual humans |  | 8 (8%) | 1 | 1 | 2 | 1 | 0 | 0 | 0 |
|  | Augmentation of clinical data |  | 9 (10%) | 5 | 0 | 4 | 0 | 4 | 2 | 1 |
| Assisting in mental health counseling and education | Counseling assistance | PERMA, Reddit, DailyDialog, EmpatheticDialogues, etc. | 9 (10%) | 0 | 0 | 2 | 0 | 0 | 0 | 0 |
|  | Mental health resource supplement |  | 2 (2%) | 0 | 0 | 0 | 0 | 0 | 0 | 0 |

Notes: DAIC: extended daic; DAIC-WOZ: distress analysis interview corpus; DTR: depressive tweets repository; USPTO: United States patent and trade-mark office; PERMA: positive emotions, engagement, relationships, meaning, and accomplishment.
