# Supplementary material for: The Applications of Large Language Models in Mental Health: Scoping Review
Source: J Med Internet Res. 2025 May 5;27:e69284. doi: 10.2196/69284 (PMC12089884; doi:10.2196/69284)
Supplement: Multimedia Appendix 7 [file jmir_v27i1e69284_app7.docx]

Supplemental Files

Table S6. The metrics results of performance between LLMs and humans.

| **Studies** | **Applications** | **Data Information** | | **Performance** | | | | | | | | | | | **Conclusion** |  |
| --- | --- | --- | --- | --- | --- | --- | --- | --- | --- | --- | --- | --- | --- | --- | --- | --- |
|  |  | **Sample size** | **Evaluation of Professional group(s)** | **Models &Human** | **Metric 1 &Performance** | | **Metric 2 &Performance** | | **Metric 3 &Performance** | | **Metric 4 &Performance** | | **Metric 5 &Performance** | |  |  |
| 1 | suicide detection and classification | 460 patients | Psychiatrists and psychologists | GPT-4 | Accuracy | Data 1^a^: 0.67 Data 2: 0.64 Data 3: 0.61 Data 4: 0.60 | Sensitivity | Data 1: 0.62 Data 2: 0.84 Data 3: 0.46 Data 4: 0.74 | Specificity | Data 1: 0.71 Data 2: 0.51 Data 3: 0.71 Data 4: 0.51 | Precision | Data 1: 0.60 Data 2: 0.54 Data 3: 0.48 Data 4: 0.48 |  | | Overall outputs: GPT-4=average clinician (almost) |  |
|  |  |  |  |  |  |  |  |  |  |  |  |  |  |  |  |  |
|  |  |  |  |  |  |  |  |  |  |  |  |  |  |  |  |  |
|  |  |  |  |  |  |  |  |  |  |  |  |  |  |  |  |  |
|  |  |  |  | Human |  | Data 1: 0.70 Data 2: 0.75 Data 3: 0.66 Data 4: 0.71 |  | Data 1: 0.53 Data 2: 0.59 Data 3: 0.40 Data 4: 0.46 |  | Data 1: 0.82 Data 2: 0.86 Data 3: 0.82 Data 4: 0.86 |  | Data 1: 0.70 Data 2: 0.77 Data 3: 0.50 Data 4: 0.69 |  |  |  |  |
|  |  |  |  |  |  |  |  |  |  |  |  |  |  |  |  |  |
|  |  |  |  |  |  |  |  |  |  |  |  |  |  |  |  |  |
|  |  |  |  |  |  |  |  |  |  |  |  |  |  |  |  |  |
| 2 | Supporting the clinical treatments and interventions | 50 sets of clinical vignettes | Experts and prescribing clinicians | GPT-4  with guideline knowledge | Top 1^b^ | 50.80% | Top 3 | 84.40% | Top 5 | 94.90% | Poor choice | 12.00% | Cohen’s kappa | 0.31 | GPT-4 with treatment guidelines > GPT-4 > Human |  |
|  |  |  |  | GPT-4 |  | 23.40% |  | 72.40% |  | 90.60% |  | 10.80% |  | 0.09 |  |  |
|  |  |  |  | Human |  | 23% |  | 49% |  | 58.40% |  | 22% |  | 0.07 |  |  |
| 3 | Develop chatbots for assisted therapy | 100 patient consultation samples and 239 questions | Chief physicians | ChatGPT-4 | Relevance | 3.75 | Accuracy | 3.73 | Usefulness | 3.4 | Empathy | 3.64 |  | | Usefulness: Human>LLM empathy: LLM>Huma, particularly ChatGPT-4 |  |
|  |  |  |  | ERNIE Bot |  | 3.41 |  | 3.52 |  | 3.05 |  | 3.11 |  |  |  |  |
|  |  |  |  | Human |  | 3.69 |  | 3.66 |  | 3.54 |  | 3.13 |  |  |  |  |
| 4 | Supporting the clinical treatments and interventions | 8 vignettes,  each repeated 10 times | Primary care physicians | ChatGPT-3.5 | Treatment recommendations for mild depression | Treatment 1^c^ (95%) Treatment 1 + Treatment 2 (5%) | Treatment recommendations for severe depression: | Treatment 1 (28%) Treatment 1 + Treatment 2 (72%) | Pharmacological approach | Drugs 1 (74%) Drugs 1 + Drugs 2 (26%) |  | |  | | Precision： GPT-4>GPT-3.5 >Primary care physicians pros of LLMs: no discernible biases related to gender and SES |  |
|  |  |  |  | ChatGPT-4 |  | Treatment 1 (97.5%) Treatment 1 + Treatment 2 (2.5%) |  | Treatment 1 + Treatment 2 (100%) |  | Drugs 1 (68%) Drugs 1 + Drugs 2 (32%) |  |  |  |  |  |  |
|  |  |  |  | Human |  | Treatment 1 (4.3%) Treatment 2 (48.3%) Treatment 1 + Treatment 2 (32.5%) None of treatments (14.9%) |  | Treatment 1 (NR) Treatment 2 (40%) Treatment 1 + Treatment 2 (44.4%) None of treatments (NR) |  | Drugs 1 (17.7%) Drugs 2 (14.0%) Drugs 1 + Drugs 2 (67.4%) None of drugs(9%) |  |  |  |  |  |  |
| 5 | Emotion interpretation | 36 photos; 20 questions | the general population | ChatGPT-4 | RMET Scores | 1st evaluation:26 2nd evaluation:27 | LEAS Scores | Total: 97 MC^d^: 79 OC: 77 |  |  |  |  |  |  | RMET Scores: ChatGPT-4=Human>  Google  Bard LEAS Scores: ChatGPT-4>Google Bard>Human |  |
|  |  |  |  |  |  |  |  |  |  |  |  |  |  |  |  |  |
|  |  |  |  | Google  Bard |  | 1st evaluation:10 2nd evaluation:12 |  | Total: 97 MC: 79 OC: 75 |  |  |  |  |  |  |  |  |
|  |  |  |  | Human |  | Mean:26.2 |  | (French  Men/Women)  Total: 56.21/58.94 MC: 49.24/53.94 OC: 46.03/48.73 |  |  |  |  |  |  |  |  |
| 6 | Supporting the clinical treatments and interventions | 19 OCD vignettes,  7 control vignettes | APA members,  primary care physicians,  doctoral trainees in psychology,  medical providers,  clergy members | ChatGPT-4 | Diagnostic accuracy | 100% |  |  |  |  |  |  |  |  | ChatGPT-4>Llama 3>Gemini Pro>Human |  |
|  |  |  |  | Gemini Pro |  | 93.80% |  |  |  |  |  |  |  |  |  |  |
|  |  |  |  | Llama 3 |  | 94.70% |  |  |  |  |  |  |  |  |  |  |
|  |  |  |  | Human |  | Group 1^e^: 61.6% Group 2: 49.5% Group 3: 81.5% Group 4: 41.9% Group 5: 35.5% |  |  |  |  |  |  |  |  |  |  |

^a^Data1: SI with plan at intake (n=140) versus no SI with plan (n=200) and Chief complaint text only; Data2: SI with plan at intake (n=140) versus no SI with plan (n=200) and Chief complaint text + previous suicide attempt knowledge; Data3: SI with plan post intake (n=120) versus no SI with plan (n=200) and Chief complaint text only; Data4: SI with plan post intake (n=120) versus no SI with plan (n=200) and Chief complaint text + previous suicide attempt knowledge.

^b^Poor choice: Sample proportion of poor or contraindicated medications; Top 1/3/5: Sample proportion of the first 1/3/5 plans with optimal decisions.

^c^Treatment 1: referral for psychotherapy; Treatment 2: prescription of pharmacological treatment; Drugs 1: antidepressants; Drugs 2: anxiolytic/hypnotic. NR: not reported.

^d^MC: main character; OC: other character.

^e^Group 1: APA members; Group 2: Primary care physicians; Group 3: Doctoral trainees in psychology; Group 4: Medical providers in Guam; Group 5: Clergy members in Guam.

References

1. Lee C, Mohebbi M, O'Callaghan E, Winsberg M. Large Language Models Versus Expert Clinicians in Crisis Prediction Among Telemental Health Patients: Comparative Study. JMIR Ment Health. 2024;11:e58129.

2. Perlis RH, Goldberg JF, Ostacher MJ, Schneck CD. Clinical decision support for bipolar depression using large language models. Neuropsychopharmacology : official publication of the American College of Neuropsychopharmacology. 2024;49(9):1412-6.

3. He W, Zhang W, Jin Y, Zhou Q, Zhang H, Xia Q. Physician Versus Large Language Model Chatbot Responses to Web-Based Questions From Autistic Patients in Chinese: Cross-Sectional Comparative Analysis. Journal of medical Internet research. 2024;26:e54706.

4. Levkovich I, Elyoseph Z. Suicide Risk Assessments Through the Eyes of ChatGPT-3.5 Versus ChatGPT-4: Vignette Study. JMIR Ment Health. 2023;10:e51232.

5. Elyoseph Z, Refoua E, Asraf K, Lvovsky M, Shimoni Y, Hadar-Shoval D. Capacity of Generative AI to Interpret Human Emotions From Visual and Textual Data: Pilot Evaluation Study. JMIR Ment Health. 2024;11:e54369.

6. Kim J, Leonte KG, Chen ML, Torous JB, Linos E, Pinto A, et al. Large language models outperform mental and medical health care professionals in identifying obsessive-compulsive disorder. NPJ Digit Med. 2024;7(1):193.
