# Supplementary material for: The Applications of Large Language Models in Mental Health: Scoping Review
Source: J Med Internet Res. 2025 May 5;27:e69284. doi: 10.2196/69284 (PMC12089884; doi:10.2196/69284)
Supplement: Multimedia Appendix 8 [file jmir_v27i1e69284_app8.docx]

Supplemental Files

Table S7. Performance comparisons between various LLMs.

| **Applications** | **Paper information** | | | **Model Information** | | | | | | | | | | | | | **Conclusions** |
| --- | --- | --- | --- | --- | --- | --- | --- | --- | --- | --- | --- | --- | --- | --- | --- | --- | --- |
|  | **Reference** | **Intended task** | **Sample size** | **Models** | **Metric1 & Performance** | | **Metric2 & Performance** | | **Metric3 & Performance** | | **Metric4 & Performance** | | **Metric5 & Performance** | | **Metric6 & Performance** | | **Evaluation results** |
| **Depression detection and classification** | [1] | Predict mental health disorders | 11,890,632 tweets | DBUFS2E | ACC for Tweets | 97% | F1 Score for Tweets | 0.96 | AUC for Tweets | 0.98 | ACC for Bios | 95% | F1 Score for Bios | 0.96 | AUC for Bios | 0.96 | DBUFS2E, BBU, MBBU > DRB |
|  |  |  |  | BBU |  | 97% |  | 0.97 |  | 0.98 |  | 95% |  | 0.94 |  | 0.96 |  |
|  |  |  |  | MBBU |  | 96% |  | 0.96 |  | 0.98 |  | 96% |  | 0.96 |  | 0.96 |  |
|  |  |  |  | DRB |  | 95% |  | 0.95 |  | 0.97 |  | 95% |  | 0.95 |  | 0.96 |  |
|  | [2] | Identify depression | 2,575 users with tweets | XLNet | Precision | 0.775 | F1 Score | 0.779 | Recall | 0.783 | ACC | 77% | AUC | 0.844 |  | | XLNet, RoBERTa > BERT |
|  |  |  |  | RoBERTa |  | 0.744 |  | 0.78 |  | 0.819 |  | 76% |  | 0.841 |  |  |  |
|  |  |  |  | BERT |  | 0.763 |  | 0.775 |  | 0.788 |  | 77% |  | 0.839 |  |  |  |
|  | [3] | Detect depression | 632,000 tweets | RoBERTa | Precision | 98% | F1 Score | 98% | Recall | 99% | ACC | 98% |  | |  | | RoBERTa > DeBERTa, DistilBERT, SqueezeBERT |
|  |  |  |  | DeBERTa |  | 98% |  | 98% |  | 98% |  | 98% |  |  |  |  |  |
|  |  |  |  | DistilBERT |  | 98% |  | 97% |  | 98% |  | 97% |  |  |  |  |  |
|  |  |  |  | SqueezeBERT |  | 97% |  | 96% |  | 97% |  | 95% |  |  |  |  |  |
|  | [4] | Detect depression | DAIC, E-DAIC, and EATD | GPT-3.5 | Precision | 65.7% | F1 Score | 60.6% | Recall | 58.9% | ACC | 58.9% |  | |  | | LLAMA 3 > GPT-4 > GPT-3.5 |
|  |  |  |  | GPT-4 |  | 71.5% |  | 71.4% |  | 71.4% |  | 71.4% |  |  |  |  |  |
|  |  |  |  | LLAMA 3 |  | 81.3% |  | 74.0% |  | 73.2% |  | 73.2% |  |  |  |  |  |
|  | [5] | Detect depression | DAIC-WOZ dataset | BERT | Precision | 89.1% | F1 Score | 88.9% | Recall | 88.9% |  | |  | |  | | ClinicalBERT > PsychBERT > MentalBERT > BERT > MentalRoBERTa |
|  |  |  |  | MentalBERT |  | 90.1% |  | 90.1% |  | 89.9% |  |  |  |  |  |  |  |
|  |  |  |  | MentalRoBERTa |  | 74.1% |  | 75.1% |  | 74.9% |  |  |  |  |  |  |  |
|  |  |  |  | PsychBERT |  | 91.1% |  | 92.9% |  | 91.1% |  |  |  |  |  |  |  |
|  |  |  |  | ClinicalBERT |  | 96.3% |  | 95.5% |  | 95.5% |  |  |  |  |  |  |  |
|  | [6] | Detect depression | 8,194 records | BERT | Precision | 0.83 | F1 score | 0.82 | Recall | 0.82 |  | |  | |  | | BERT > GPT-3.5, GPT-4 |
|  |  |  |  | GPT-3.5 |  | 0.78 |  | 0.78 |  | 0.79 |  |  |  |  |  |  |  |
|  |  |  |  | GPT-4 |  | 0.7 |  | 0.61 |  | 0.6 |  |  |  |  |  |  |  |
|  | [7] | Predict depression | 13,993 microblogs | RoBERTa | Macro Precision | 0.528 | Macro F1 Score | 0.424 | Macro Recall | 0.386 |  | |  | |  | | BERT, RoBERTa > XLNET |
|  |  |  |  | BERT |  | 0.543 |  | 0.406 |  | 0.354 |  |  |  |  |  |  |  |
|  |  |  |  | XLNET |  | 0.521 |  | 0.398 |  | 0.364 |  |  |  |  |  |  |  |
|  | [8] | Detect depression symptoms | Posts of 9,210 depressed users | BERT | Positive Predictive Value | 0.638 | F1 Score | 0.709 | Sensitivity | 0.805 |  | |  | |  | | BioBERT, MentalBERT > BERT, ALBERT, Longformer, MentalRoBERTa |
|  |  |  |  | ALBERT |  | 0.606 |  | 0.683 |  | 0.786 |  |  |  |  |  |  |  |
|  |  |  |  | BioBERT |  | 0.601 |  | 0.707 |  | 0.862 |  |  |  |  |  |  |  |
|  |  |  |  | Longformer |  | 0.633 |  | 0.719 |  | 0.838 |  |  |  |  |  |  |  |
|  |  |  |  | MentalBERT |  | 0.66 |  | 0.738 |  | 0.848 |  |  |  |  |  |  |  |
|  |  |  |  | MentalRoBERTa |  | 0.629 |  | 0.709 |  | 0.819 |  |  |  |  |  |  |  |
|  | [9] | Classify depression | 189 interviews | BERT | F1 Score | 0.6 | Sensitivity | 0.61 | Specificity | 0.6 |  | |  | |  | | BERT, DistilBERT > RoBERTa |
|  |  |  |  | RoBERTa |  | 0.47 |  | 0.54 |  | 0.34 |  |  |  |  |  |  |  |
|  |  |  |  | DistilBERT |  | 0.59 |  | 0.59 |  | 0.62 |  |  |  |  |  |  |  |
|  | [10] | Classify depression and anxiety | GLOBEM dataset | PaLM2 | ACC of depression | 60.0% | ACC of anxiety | 56.7% |  | |  | |  | |  | | PaLM2 > GPT-4 > GPT-3.5 |
|  |  |  |  | GPT-4 |  | 57.8% |  | 55.6% |  |  |  |  |  |  |  |  |  |
|  |  |  |  | GPT-3.5 |  | 56.7% |  | 52.5% |  |  |  |  |  |  |  |  |  |
|  | [11] | Handle PPD questions | 14 questions | ChatGPT | Mean of GRADE | 3.93 | Median of GRADE | 4 |  | |  | |  | |  | | ChatGPT > Bard |
|  |  |  |  | Bard |  | 2.75 |  | 4 |  |  |  |  |  |  |  |  |  |
| Developing conversational virtual humans | [12] | Assess efficacy of chatbots | Unreported | ChatGPT | Safety | 4.0 | Validation | 3.9 | Consistency | 4.1 | Empathy | 3.9 | Insight | 4.2 |  | | ChatGPT > Claude-3 > GPT-4 |
|  |  |  |  | GPT-4 |  | 3.7 |  | 4.1 |  | 3.5 |  | 3.6 |  | 3.7 |  |  |  |
|  |  |  |  | Claude-3 |  | 3.8 |  | 4.1 |  | 3.8 |  | 3.7 |  | 3.8 |  |  |  |
|  | [13] | Assess LLM chatbots | 100 patients and 239 questions | Physicians | Prefer Rate | 46.9% | Relevance | 3.75 | Correct | 3.66 | Useful | 3.54 | Empathy | 3.13 |  | | ChatGPT > ERNIEBot |
|  |  |  |  | ChatGPT |  | 34.9% |  | 3.69 |  | 3.73 |  | 3.4 |  | 3.64 |  |  |  |
|  |  |  |  | ERNIEBot |  | 18.3% |  | 3.41 |  | 3.52 |  | 3.4 |  | 3.11 |  |  |  |
| Supporting the clinical treatments and interventions | [14] | Utilize EHRs | 290,482,002 clinical notes | BioBERT | Precision of Clinical Concept | 0.86 | Recall of Clinical Concept | 0.89 | F1 score of Clinical Concept | 0.88 | Precision of Medical Relation | 0.97 | Recall of Medical Relation | 0.95 | F1 score of Medical Relation | 0.96 | GatorTron > BioBERT, ClinicalBERT, BioMegatron |
|  |  |  |  | ClinicalBERT |  | 0.86 |  | 0.88 |  | 0.87 |  | 0.97 |  | 0.94 |  | 0.95 |  |
|  |  |  |  | BioMegatron |  | 0.87 |  | 0.89 |  | 0.88 |  | 0.97 |  | 0.94 |  | 0.96 |  |
|  |  |  |  | GatorTron |  | 0.90 |  | 0.90 |  | 0.90 |  | 0.98 |  | 0.95 |  | 0.96 |  |
|  | [15] | Handle CBT tasks | 3 stages | GPT-4 | ACC of Stage 1 | 65% | ACC of Stage 2 | 75% | ACC of Stage 3 | 80% | ACC of All | 73% |  | |  | | GPT-4 > BARD |
|  |  |  |  | Bard |  | 65% |  | 50% |  | 95% |  | 70% |  |  |  |  |  |
|  | [16] | Assist treatment of CB-PTSD | 1,295 women narratives | GPT-3.5-turbo zero shot | F1 Score | 0.33 | Sensitivity | 0.20 | Specificity | 0.99 | AUC | 0.60 |  | |  | | ChatGPT > GPT-3.5-turbo few shot > GPT-3.5-turbo zero shot |
|  |  |  |  | GPT-3.5-turbo few shot |  | 0.38 |  | 0.24 |  | 0.96 |  | 0.60 |  |  |  |  |  |
|  |  |  |  | ChatGPT |  | 0.81 |  | 0.85 |  | 0.75 |  | 0.80 |  |  |  |  |  |
|  | [17] | Evaluate patients’ satisfaction | 16,950 illness categories | BERT | ACC | 84.6% | F1 Score | 0.846 | K-coefficient | 0.746 |  | |  | |  | | MentalBERT > BERT |
|  |  |  |  | MentalBERT |  | 86.7% |  | 0.867 |  | 0.785 |  |  |  |  |  |  |  |
|  | [18] | Test depression diagnosis | Unreported | LLamA-7B | Safety | 7.31 | Usability | 3.97 | Fluency | 9.78 |  | |  | |  | | ChatGLM-6B, Knowledge > Alpaca, LLamA-7B |
|  |  |  |  | ChatGLM-6B |  | 7.03 |  | 3.34 |  | 9.89 |  |  |  |  |  |  |  |
|  |  |  |  | Alpaca |  | 7.44 |  | 4.31 |  | 9.85 |  |  |  |  |  |  |  |
|  |  |  |  | Knowledge |  | 9.58 |  | 7.74 |  | 9.75 |  |  |  |  |  |  |  |
| Assisting in mental health counseling and education | [19] | Develop a therapeutic chatbot | 13k and 25k dialogues | BERT-based | ACC of Sentiment | >90% | ACC of Emotion | >80% |  | |  | |  | |  | | BERT-based > Bayes-based, FastTex-based |
|  |  |  |  | Bayes-based |  | —— |  | —— |  |  |  |  |  |  |  |  |  |
|  |  |  |  | FastTex-based |  | —— |  | —— |  |  |  |  |  |  |  |  |  |
|  | [20] | Enhance emotional intelligence | 31 unique questions | ChatGLM | Mean Rank | 47.00 | Rank Sum | 1457 |  | |  | |  | |  | | ChatGLM > Qianwen > ERNIEBot |
|  |  |  |  | ERNIEBot |  | 46.61 |  | 1445 |  |  |  |  |  |  |  |  |  |
|  |  |  |  | Qianwen |  | 46.87 |  | 1453 |  |  |  |  |  |  |  |  |  |
|  | [21] | Enhance diagnostic accuracy in psychiatry | 20 distinct clinical cases | AYA | ACC Mean Score | 0.95 |  | |  | |  | |  | |  | | GPT3.5, GPT4, GPT3.5 CA > AYA, Nemotron, Nemotron CA |
|  |  |  |  | GPT-3.5 |  | 1.65 |  |  |  |  |  |  |  |  |  |  |  |
|  |  |  |  | GPT-4 |  | 1.60 |  |  |  |  |  |  |  |  |  |  |  |
|  |  |  |  | Nemotron |  | 1.10 |  |  |  |  |  |  |  |  |  |  |  |
|  |  |  |  | GPT-3.5 CA |  | 1.55 |  |  |  |  |  |  |  |  |  |  |  |
|  |  |  |  | Nemotron CA |  | 1.05 |  |  |  |  |  |  |  |  |  |  |  |
| Others | [22] | Rate emotion in psycho-therapy | 97,497 talk turns | BERT | ACC | 66% | Kappa Neutral | 0.73 | Kappa Positive | 0.47 | Kappa Negative | 0.59 | Kappa Estimate | 0.48 |  | | BERT > Trigram > Bigram, Unigram |
|  |  |  |  | Unigram |  | 60% |  | 0.71 |  | 0.34 |  | 0.45 |  | 0.31 |  |  |  |
|  |  |  |  | Bigram |  | 60% |  | 0.71 |  | 0.34 |  | 0.45 |  | 0.31 |  |  |  |
|  |  |  |  | Trigram |  | 61% |  | 0.71 |  | 0.34 |  | 0.45 |  | 0.31 |  |  |  |
|  | [23] | Evaluate LLMs in psychiatric diagnosis | 13 case scenarios | GPT-3.5 | QA | 100% | Patient Doctor | 80% | Diary | 100% | USMLE | 0.51 |  | |  | | GPT-4 > GPT-3.5 > AYA > Nemotron |
|  |  |  |  | AYA |  | 100% |  | 80% |  | 100% |  | 0.24 |  |  |  |  |  |
|  |  |  |  | Nemotron |  | 80% |  | 40% |  | 67% |  | 0.19 |  |  |  |  |  |
|  |  |  |  | GPT-4 |  | —— |  | —— |  | —— |  | 0.80 |  |  |  |  |  |
|  | [24] | Analyse sentiment | NIH dataset with 462,518 tokens and stanford dataset with 299,735 tokens | Pysentimiento on NIH dataset | Precision | 77.4% | Recall | 77.8% | F1 score | 77.0% | ACC | 77.6% |  | |  | | ChatGPT > OPT-1.3B, OPT-2.7B > Pysentimiento |
|  |  |  |  | OPT-1.3B on NIH dataset |  | 80.3% |  | 80.0% |  | 79.9% |  | 80.0% |  |  |  |  |  |
|  |  |  |  | OPT-2.7B on NIH dataset |  | 80.6% |  | 80.4% |  | 80.5% |  | 80.4% |  |  |  |  |  |
|  |  |  |  | ChatGPT on NIH dataset |  | 85.3% |  | 89.3% |  | 86.7% |  | 86.0% |  |  |  |  |  |
|  |  |  |  | Pysentimiento on Standford dataset |  | 67.3% |  | 63.6% |  | 62.7% |  | 64.4% |  |  |  |  |  |
|  |  |  |  | OPT-1.3B on Standford dataset |  | 83.2% |  | 81.6% |  | 82.1% |  | 81.6% |  |  |  |  |  |
|  |  |  |  | OPT-2.7B on Standford dataset |  | 82.9% |  | 81.0% |  | 81.5% |  | 81.0% |  |  |  |  |  |
|  |  |  |  | ChatGPT on Standford dataset |  | 86.3% |  | 87.8% |  | 86.6% |  | 87.4% |  |  |  |  |  |
|  | [25] | Evaluate LLMs for mental health condition detection | 2.8k stress posts, 9.21k depression posts and 1.78k suicide posts from social media like Reddit and Twitter | GPT-2 on stress data | Precision | 98.4% | Recall | 98.4% | F1 score | 98.4% | ACC | 98.4% |  | |  | | GPT-2 > GPT-Neo |
|  |  |  |  | GPT-Neo on stress data |  | 77.6% |  | 76.5% |  | 77.6% |  | 77.2% |  |  |  |  |  |
|  |  |  |  | GPT-2 on depression data |  | 98.9% |  | 98.9% |  | 98.8% |  | 99.0% |  |  |  |  |  |
|  |  |  |  | GPT-Neo on depression data |  | 94.5% |  | 94.4% |  | 94.4% |  | 94.5% |  |  |  |  |  |
|  |  |  |  | GPT-2 on suicide data |  | 99.7% |  | 99.8% |  | 99.7% |  | 98.7% |  |  |  |  |  |
|  |  |  |  | GPT-Neo on suicide data |  | 93.5% |  | 94.6% |  | 94.0% |  | 94.4% |  |  |  |  |  |
|  | [26] | Identify emotions and sarcasm | 43,410 Reddit comments | Custom BERT | Validation ACC | 59.5% | Validation Loss | 1.07 | Testing ACC | 61.7% | Testing Loss | 1.07 |  | |  | | Mobile BERT > Small Size BERT > Custom BERT |
|  |  |  |  | Small Size BERT |  | 61.3% |  | 1.04 |  | 64.2% |  | 1.00 |  |  |  |  |  |
|  |  |  |  | Mobile BERT |  | 69.6% |  | 0.81 |  | 74.5% |  | 0.66 |  |  |  |  |  |
|  | [27] | Classify emotion  and sentiment | 417,423 and 1,176,509 samples | EmoBERTTiny | ACC of Sentiment | 93.1% | ACC of Emotion | 85.5% |  | |  | |  | |  | | EmoBERTTiny > BERTTiny > Bert-Base Cased |
|  |  |  |  | BERTTiny |  | 38.1% |  | 14.7% |  |  |  |  |  |  |  |  |  |
|  |  |  |  | Bert-Base Cased |  | 30.4% |  | 8.2% |  |  |  |  |  |  |  |  |  |
|  | [28] | Detect suicidal ideation | 100,000+ posts | ALBERT | ACC | 87% | F1 Score | 0.87 |  | |  | |  | |  | | ALBERT > DistilBERT |
|  |  |  |  | DistilBERT |  | 77% |  | 0.75 |  |  |  |  |  |  |  |  |  |
|  | [29] | Generate synthetic medical data | 189 real and 200 synthetic patients | ChatGPT | ACC | 89% | ACC of complex data | 77% |  | |  | |  | |  | | ChatGPT > Bard |
|  |  |  |  | Bard |  | 87% |  | 74% |  |  |  |  |  |  |  |  |  |
|  | [30] | Diagnose OCD | 19 OCD and 7 control vignettes | GPT-4 | ACC | 1.00 |  | |  | |  | |  | |  | | GPT-4 > GEMINI PRO > LLAMA 3 |
|  |  |  |  | GEMINI PRO |  | 0.94 |  |  |  |  |  |  |  |  |  |  |  |
|  |  |  |  | LLAMA 3 |  | 0.95 |  |  |  |  |  |  |  |  |  |  |  |
|  | [31] | Analyze psychologic text | 47,925 tweets of 15 datasets | GPT-3.5 Turbo | SROCC | 0.63 |  | |  | |  | |  | |  | | GPT-4 > GPT-4 Turbo > GPT-3.5 Turbo |
|  |  |  |  | GPT-4 |  | 0.69 |  |  |  |  |  |  |  |  |  |  |  |
|  |  |  |  | GPT-4 Turbo |  | 0.67 |  |  |  |  |  |  |  |  |  |  |  |

Notes:

ACC: accuracy; AUC: area under curve; BBU: bert-base-uncased; MBBU: mental-bert-base-uncased; CB-PTSD: post-traumatic stress disorder following childbirth; CBT: cognitive-behavioral therapy; DBUFS2E: distilbert-base-uncased-finetuned-sst-2-english; DRB: distilroberta-base; EHR: electronic health records; GRADE: grading of recommendations assessment, development and evaluation; OCD: obsessive-compulsive disorder; PPD: postpartum depression; USMLE: United Stated medical licensing exam.

References

1. Pourkeyvan A, Safa R, Sorourkhah A. Harnessing the Power of Hugging Face Transformers for Predicting Mental Health Disorders in Social Networks. IEEE Access. 2024;12:28025-35.

2. Zhang Y, Lyu H, Liu Y, Zhang X, Wang Y, Luo J. Monitoring Depression Trends on Twitter During the COVID-19 Pandemic: Observational Study. JMIR Infodemiology. 2021;1(1):e26769.

3. Bokolo BG, Liu Q. Deep Learning-Based Depression Detection from Social Media: Comparative Evaluation of ML and Transformer Techniques. ELECTRONICS. 2023;12(21).

4. Sood P, Yang X, Wang P, editors. Enhancing Depression Detection from Narrative Interviews Using Language Models. 2023 IEEE International Conference on Bioinformatics and Biomedicine (BIBM); 2023 5-8 Dec. 2023.

5. Firoz N, Berestneva O, Aksyonov SV, editors. Dual Layer Cogni - Insight Deep-Mood Encoder: A Two- Tiered Approach for Depression Detection. 2024 International Russian Smart Industry Conference (SmartIndustryCon); 2024 25-29 March 2024.

6. Danner M, Hadzic B, Gerhardt S, Ludwig S, Uslu I, Shao P, et al., editors. Advancing Mental Health Diagnostics: GPT-Based Method for Depression Detection. 2023 62nd Annual Conference of the Society of Instrument and Control Engineers (SICE); 2023 6-9 Sept. 2023.

7. Wang X, Chen S, Li T, Li W, Zhou Y, Zheng J, et al. Depression Risk Prediction for Chinese Microblogs via Deep-Learning Methods: Content Analysis. JMIR medical informatics. 2020;8(7):e17958.

8. Owen D, Antypas D, Hassoulas A, Pardinas AF, Espinosa-Anke L, Collados JC. Enabling Early Health Care Intervention by Detecting Depression in Users of Web-Based Forums using Language Models: Longitudinal Analysis and Evaluation. Jmir ai. 2023;2:e41205.

9. Senn S, Tlachac ML, Flores R, Rundensteiner E, editors. Ensembles of bert for depression classification. 2022 44th Annual International Conference of the IEEE Engineering in Medicine & Biology Society (EMBC); 2022: IEEE.

10. Englhardt Z, Ma C, Morris ME, Chang CC, Xu XO, Qin L, et al. From Classification to Clinical Insights: Towards Analyzing and Reasoning About Mobile and Behavioral Health Data With Large Language Models. Proceedings of the ACM on Interactive, Mobile, Wearable and Ubiquitous Technologies -IMWUT. 2024;8(2).

11. Sezgin E, Chekeni F, Lee J, Keim S. Clinical Accuracy of Large Language Models and Google Search Responses to Postpartum Depression Questions: Cross-Sectional Study. Journal of medical Internet research. 2023;25:e49240.

12. Berrezueta-Guzman S, Kandil M, Martin-Ruiz ML, de la Cruz IP, Krusche S, Ieee Computer SOC. Exploring the Efficacy of Robotic Assistants with ChatGPT and Claude in Enhancing ADHD Therapy: Innovating Treatment Paradigms. 20th International Conference on Intelligent Environments, IE 2024; 20242024. p. 25-32.

13. He W, Zhang W, Jin Y, Zhou Q, Zhang H, Xia Q. Physician Versus Large Language Model Chatbot Responses to Web-Based Questions From Autistic Patients in Chinese: Cross-Sectional Comparative Analysis. Journal of medical Internet research. 2024;26:e54706.

14. Yang X, Chen A, PourNejatian N, Shin HC, Smith KE, Parisien C, et al. A large language model for electronic health records. NPJ Digit Med. 2022;5(1):194.

15. Hodson N, Williamson S. Can Large Language Models Replace Therapists? Evaluating Performance at Simple Cognitive Behavioral Therapy Tasks. Jmir ai. 2024;3:e52500.

16. Bartal A, Jagodnik KM, Chan SJ, Dekel S. AI and narrative embeddings detect PTSD following childbirth via birth stories. Scientific reports. 2024;14(1):8336.

17. Wang Y, Yu Y, Liu Y, Ma Y, Pang PC. Predicting Patients' Satisfaction With Mental Health Drug Treatment Using Their Reviews: Unified Interchangeable Model Fusion Approach. JMIR Ment Health. 2023;10:e49894.

18. Wang X, Liu K, Wang C, editors. Knowledge-enhanced Pre-training large language model for depression diagnosis and treatment. 2023 IEEE 9th International Conference on Cloud Computing and Intelligent Systems (CCIS); 2023 12-13 Aug. 2023.

19. Zygadlo A, editor A Therapeutic Dialogue Agent for Polish Language. 2021 9th International Conference on Affective Computing and Intelligent Interaction Workshops and Demos (ACIIW); 2021 28 Sept.-1 Oct. 2021.

20. Huang S, Fu F, Yang K, Zhang K, Yang F, editors. Empowerment of Large Language Models in Psychological Counseling through Prompt Engineering. 2024 IEEE 4th International Conference on Software Engineering and Artificial Intelligence (SEAI); 2024 21-23 June 2024.

21. Gargari OK, Fatehi F, Mohammadi I, Firouzabadi SR, Shafiee A, Habibi G. Diagnostic accuracy of large language models in psychiatry. Asian J Psychiatr. 2024;100:104168.

22. Tanana MJ, Soma CS, Kuo PB, Bertagnolli NM, Dembe A, Pace BT, et al. How do you feel? Using natural language processing to automatically rate emotion in psychotherapy. Behavior research methods. 2021;53(5):2069-82.

23. Gargari OK, Habibi G, Nilchian N, Farzan AS. Comparative analysis of large language models in psychiatry and mental health: A focus on GPT, AYA, and Nemotron-3-8B - 8B. Asian Journal of Psychiatry. 2024;99.

24. Lossio-Ventura JA, Weger R, Lee AY, Guinee EP, Chung J, Atlas L, et al. A Comparison of ChatGPT and Fine-Tuned Open Pre-Trained Transformers (OPT) Against Widely Used Sentiment Analysis Tools: Sentiment Analysis of COVID-19 Survey Data. JMIR Ment Health. 2024;11:e50150.

25. Jain B, Goyal G, Sharma M, editors. Evaluating Emotional Detection & Classification Capabilities of GPT-2 & GPT-Neo Using Textual Data. 2024 14th International Conference on Cloud Computing, Data Science & Engineering (Confluence); 2024 18-19 Jan. 2024.

26. Goyal T, Rajeshbai DH, Gopalkrishna N, T M, M HR, editors. Mobile Machine Learning Models for Emotion and Sarcasm Detection in Text: A Solution for Alexithymic Individuals. 2024 3rd International Conference for Innovation in Technology (INOCON); 2024 1-3 March 2024.

27. Stigall W, Al Hafiz Khan MA, Attota D, Nweke F, Pei Y. Large Language Models Performance Comparison of Emotion and Sentiment Classification. Proceedings of the 2024 ACM Southeast Conference on ZZZ2024. p. 60-8.

28. Ghanadian H, Nejadgholi I, Osman HA. Socially Aware Synthetic Data Generation for Suicidal Ideation Detection Using Large Language Models. IEEE Access. 2024;12:14350-63.

29. İ A, M K, editors. Use of large language models for medical synthetic data generation in mental illness. 7th IET Smart Cities Symposium (SCS 2023); 2023 3-5 Dec. 2023.

30. Kim J, Leonte KG, Chen ML, Torous JB, Linos E, Pinto A, et al. Large language models outperform mental and medical health care professionals in identifying obsessive-compulsive disorder. NPJ Digit Med. 2024;7(1):193.

31. Rathje S, Mirea DM, Sucholutsky I, Marjieh R, Robertson CE, Van Bavel JJ. GPT is an effective tool for multilingual psychological text analysis. Proceedings of the National Academy of Sciences of the United States of America. 2024;121(34):e2308950121.
